# A Novel Self‐Amplifying mRNA with Decreased Cytotoxicity and Enhanced Protein Expression by Macrodomain Mutations

**DOI:** 10.1002/advs.202402936

**Published:** 2024-09-23

**Authors:** Yue Gong, Danni Yong, Gensheng Liu, Jiang Xu, Jun Ding, William Jia

**Affiliations:** ^1^ Shanghai Virogin Biotech Co. Ltd Jiading District Shanghai 200000 China; ^2^ Virogin Biotech Canada Ltd Vancouver BC V6V 3A4 Canada

**Keywords:** innate immune response, protein expression, self‐amplifying RNA

## Abstract

The efficacy and safety of self‐amplifying mRNA (saRNA) have been demonstrated in COVID‐19 vaccine applications. Unlike conventional non‐replicating mRNA (nrmRNA), saRNA offers a key advantage: its self‐replication mechanism fosters efficient expression of the encoded protein, leading to substantial dose savings during administration. Consequently, there is a growing interest in further optimizing the expression efficiency of saRNA. In this study, in vitro adaptive passaging of saRNA is conducted under exogenous interferon pressure, which revealed several mutations in the nonstructural protein (NSP). Notably, two stable mutations, Q48P and I113F, situated in the NSP3 macrodomain (MD), attenuated its mono adenosine diphosphate ribose (MAR) hydrolysis activity and exhibited decreased replication but increased payload expression compared to wild‐type saRNA (wt saRNA). Transcriptome sequencing analysis unveils diminished activation of the double‐stranded RNA (dsRNA) sensor and, consequently, a significantly reduced innate immune response compared to wt saRNA. Furthermore, the mutant saRNA demonstrated less translation inhibition and cell apoptosis than wt saRNA, culminating in higher protein expression both in vitro and in vivo. These findings underscore the potential of reducing saRNA replication‐dependent dsRNA‐induced innate immune responses through genetic modification as a valuable strategy for optimizing saRNA, enhancing payload translation efficiency, and mitigating saRNA cytotoxicity.

## Graphical abstract

This research shows an example that reducing innate immunogenicity to increase payload protein expression of self‐amplifying RNA via a single mutation in the macrodomain. The mutant saRNA attenuates the activation of the double‐stranded RNA sensor as well as the downstream interferon response. It has improved ribosomal RNA stability and reduced cytotoxicity, leading to enhanced payload protein expression.

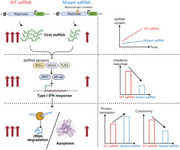

## 1 Introduction

As the next generation mRNA molecules, self‐amplifying mRNA (saRNA) has demonstrated advantages in vaccine applications due to its self‐adjuvant effect and prolonged expression duration.^[^ ^1^, ^2^, ^3^, ^4^, ^5^, ^6^ ^]^ saRNAs typically consist of three main components of RNA sequence: the sequence encoding replication machinery, a subgenomic RNA sequence encoding the payload of protein of interest, and additional non‐structural elements.^[^ ^7^ ^]^ The replication machinery includes elements derived from RNA viruses, such as viral polymerases and helicases, which enable the saRNA to replicate within host cells. Importantly, replication of RNA sequence encoding the replication machinery and the payload are differentially regulated within saRNA‐transfected cells, where more copies of the subgenomic mRNA encoding payload are produced, leading to increased expression of target proteins.^[^ ^8^ ^]^

Overall, the structure of saRNA is designed to enable efficient replication and expression of the target proteins, making it a promising platform for vaccine development and other therapeutic applications.^[^ ^9^, ^10^ ^]^ The approval of a COVID‐19 saRNA vaccine in Japan in 2023 further validates its safety profile.^[^ ^11^ ^]^ In comparison to non‐replicating mRNA (nrmRNA), a significant advantage of saRNA lies in its higher and more sustained expression of exogenous genes, leading to a reduction in administered doses. Meanwhile, the replicative nature of saRNA also raises potential safety concerns, as uncontrolled replication could lead to cellular toxicity or immune‐mediated adverse reactions.^[^ ^12^, ^13^ ^]^ Therefore, optimizing saRNA sequences to further enhance expression efficiency and safety is still a focal point.

The utilization of nucleotide modifications to evade recognition by pattern recognition receptors (PRRs) has been highly effective in reducing the innate immune response and improving gene expression efficiency in nrmRNA.^[^ ^14^, ^15^ ^]^ However, incorporating such modifications into saRNAs presents unique challenges due to the alphavirus‐derived sequences they contain. Nucleotide modification in saRNAs is often hindered by the risk of compromising the functionality of the replication machinery, thus posing a significant challenge. Furthermore, even a nucleotide modified saRNA can be viable, many copies of progeny RNA of amplification product are unmodified.^[^ ^16^, ^17^ ^]^ In addition to unmodified nucleotides, replication byproducts including double‐stranded RNA (dsRNA) intermediates, cap0‐structured progeny RNA, and capless 5′ triphosphate RNA are all recognized as pathogen‐associated molecular patterns (PAMPs) by PRRs. Upon recognition, these patterns trigger antiviral immune responses, including but not limited to the interferon (IFN) or nuclear factor kappa‐light‐chain‐enhancer of activated B cells (NF‐κB) signaling pathways, inducing abundant interferon to stimulate genes such as protein kinase R (PKR), 2′‐5′‐oligoadenylate synthetase (OAS) and interferon induced proteins with tetratricopeptide repeats (IFIT) to directly restrict saRNA replication and expression. Pepini et al. observed a significant increase in the expression of saRNA in mice lacking type I interferon receptors or in naïve mice whose type I interferon receptors were blocked by antibodies.^[^ ^18^ ^]^ Furthermore, Zhong et al. demonstrated the efficient suppression of the type I IFN response and enhanced translation of saRNA through the topical application of innate immune inhibitors.^[^ ^19^ ^]^ These findings indicate that an exaggerated innate immune response significantly inhibits the expression levels and sustained expression of payload. Hence, improved saRNA expression efficiency might be accomplished by suppressing the IFN response.

Several strategies have been reported to improve saRNA expression. One strategy introduces viral innate inhibiting proteins, either by encoding simultaneously with payload or administered concomitantly with saRNA to evade the immune response.^[^ ^20^, ^21^ ^]^ Nevertheless, the potential safety issues associated with both the virus protein itself and the robust suppression of the natural immune response require careful and thorough evaluation. Another strategy entails the introduction of alphavirus translation enhancers to boost subgenomic RNA replication or evade host translation inhibition.^[^ ^22^, ^23^ ^]^ However, potential affection for protein functionality resulting from the fusion of enhancing sequences with payload requires further confirmation.

In the present study, we have employed a strategy focused on introducing mutations into the saRNA backbone to augment expression efficiency. This approach eliminates the necessity for introducing redundant components, and its optimization effects extend across the entire saRNA replication and expression process. Given the limited understanding of alphavirus functions, particularly its intricately multifunctional non‐structural proteins, we opted to obtain mutant saRNAs through in vitro adaptive evolution screening rather than rational design. This strategy capitalizes on saRNA's high replication levels, short replication cycle, and relatively low fidelity of its RNA‐dependent RNA polymerase, resulting in a high mutation frequency overall. Therefore, serial passaging in vitro emerges as a rapid method to acquire adaptive mutations. Moreover, recognizing the critical role of the interferon response in impacting saRNA expression efficiency, we strategically incorporated interferon as a selection pressure to facilitate the evolution of adaptive mutations within cells. We hypothesized that these mutations could confer greater resistance to the interferon response induced by saRNA transfection, consequently enhancing the expression of the encoded payload.

## 2 Results

### 2.1 In Vitro Evolution of Adaptive Mutations Under Interferon Pressure

#### 2.1.1 Selection of Cell Lines

**Figure 1:**
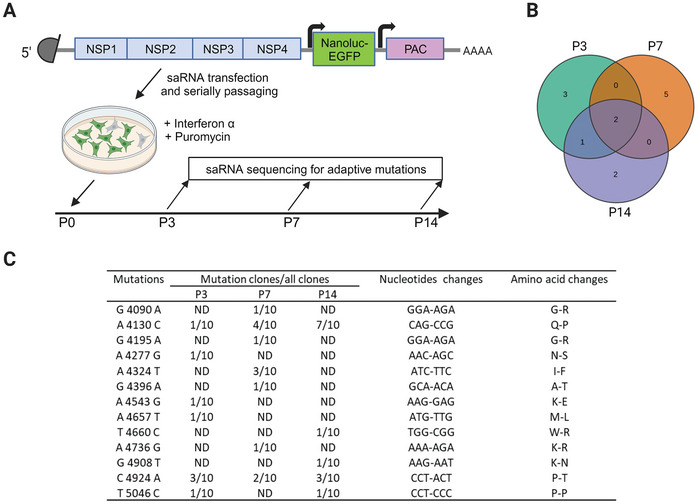
Evolution and sequencing of adaptive mutations under interferon pressure. A) Schematic diagram of the screening process for saRNA adaptive mutations. Huh7.5.1 cells pre‐treated with IFNα were subjected to transfection with saRNA co‐expressing nano luciferase fused EGFP and puromycin n‐acetyl‐transferase, then the cells were serially passaged in the medium supplemented with IFNα and puromycin. At indicated passaging, cellular RNA was extracted for adaptive mutation identification by amplicon sequencing. B) Venn diagram of the adaptive mutation numbers that appeared at different passages. C) Sequence information of adaptive mutations in saRNA at different passages, the first nucleotide of saRNA designated as 1. ND, not detected.

Utilizing the Venezuelan equine encephalitis virus (VEEV) genome sequence as a blueprint, we engineered a synthetic RNA (saRNA) encoding nano‐luciferase fused with an enhanced green fluorescent protein (EGFP) and puromycin N‐acetyl‐transferase (PAC) simultaneously (**Figure** **1**A). This RNA was synthesized through in vitro transcription and introduced into BHK‐21, Huh7.5.1, C2C12, and RAW264.7 cell lines. BHK‐21 and Huh7.5.1 cells, known for their utility in studying RNA viruses such as alphavirus and hepatitis C virus due to their permissiveness to viral replicon replication,^[^ ^24^, ^25^ ^]^ exhibited sustained EGFP expression over a 6‐day period post‐transfection (Figure S1A, Supporting Information). In contrast, C2C12 and RAW264.7 cells, which are susceptible to viral infection, particularly RAW264.7, a primary model for innate immunity research, displayed a gradual decline in EGFP expression accompanied by significant cell death.

Among these cell lines, BHK‐21 cells are insensitive to IFN due to the absence of type I IFN receptors, while Huh7.5.1 cells, with a frameshift mutation in the bcl2‐associated X (BAX) gene and deficient retinoic acid‐inducible gene I (RIG‐I) signaling transduction,^[^ ^26^ ^]^ display reduced IFN induction and cell death post‐saRNA transfection. However, pre‐treating Huh7.5.1 cells with exogenous IFNα2 before saRNA transfection induces robust endogenous IFN and downstream IFIT2 production, leading to a dose‐dependent suppression of saRNA expression (Figure S1B,C, Supporting Information). These results indicate that priming Huh7.5.1 cells with IFN before saRNA transfection effectively applies IFN‐mediated antiviral pressure, facilitating adaptive mutation screening for saRNA sequences. Moreover, concurrent puromycin treatment eliminates untransfected or poorly expressed cells, thereby enhancing screening efficiency.

#### 2.1.2 Mutants of saRNAs Selected Via Interferon‐α Treatment

Before transfection with saRNA, Huh7.5.1 cells underwent pretreatment with IFNα2 at varying concentrations: 1 pg mL^−1^, 1 ng mL^−1^, and 1 µg mL^−1^, respectively. Following transfection, cells were supplemented with 4 µg mL^−1^ puromycin after 24 h. Interestingly, only cells pretreated with 1 pg mL^−1^ of IFNα2 sustained stable replication of saRNA, while cells in the other two groups experienced rapid cell death upon puromycin addition. Consequently, cells exhibiting stable saRNA replication after pretreatment with 1 pg mL^−1^ IFNα2 were selected for serial passaging, and cellular RNA was collected from different passages. Subsequent amplicon sequencing targeting the nonstructural protein (NSP) region revealed mutations exclusively within NSP3, with a total of 11 missense mutations and 1 synonymous mutation appearing during passaging. Notably, only the nucleotide mutation frequencies of A4130C, C4924A, and A4324T exceeded 30% in specific passages, with the former two mutations consistently present across all passages (Figure 1B). Moreover, the proportion of A4130C gradually increased with passaging, reaching 70% by the 14th passage (Figure 1C), indicating the significant stability of this mutation. In summary, to bolster the saRNA‐induced innate response, we treated Huh7.5.1 cells sustaining stable saRNA replication with exogenous interferon. Subsequently, we conducted serial passaging, culminating in the identification of three adaptive mutations with relatively high frequencies for further analysis.

### 2.2 The Adaptive Mutations Show Elevated Reporter Gene Expression Along with Attenuated Replication and Decreased Cytotoxicity

#### 2.2.1 Amplification of Subgenomic RNA in Mutants

**Figure 2:**
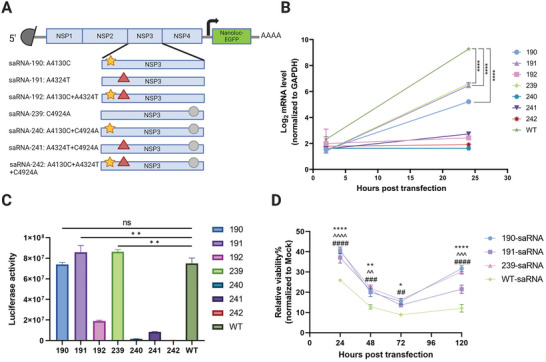
In vitro screening of adaptive mutations associated with elevated expression potency. A) Schematic of various adaptive mutations introduction in saRNA. Different shapes represent distinct adaptive mutations. B) The level of subgenomic RNA encoding EGFP in saRNA transfected Huh7.5.1 cells was assessed at 2 and 24 h. Significance was determined by two‐way ANOVA with Tukey's multiple‐comparison test. ^****^, *p* < 0.0001; C) Nano luciferase activity in the supernatant of saRNA transfected Huh7.5.1 cells were measured at 24 h. Significance was determined by one‐way ANOVA with Tukey's multiple‐comparison test. ^**^, *p* < 0.01. ns, no significance. D) Cell viability of different saRNA transfected Huh7.5.1 cells was assessed at 24, 48, 72, and 120 h. Significance was determined by two‐way ANOVA with Tukey's multiple‐comparison test. ^*^, *p* < 0.05; ^**^, *p* < 0.01; ^****^, *p* < 0.0001 (WT versus 190). ^^^^, *p* < 0.01; ^^^^^, *p* < 0.001; ^^^^^^, *p* < 0.0001 (WT versus 191). ^##^, *p* < 0.01; ^###^, *p* < 0.001; ^####^, *p* < 0.0001 (WT versus 239).

The three adaptive mutations were combined and introduced into the wt saRNA, resulting in the generation of seven saRNA variants carrying nano luciferase fused EGFP as the payload (**Figure** **2**A). We evaluated the replicative capacity of the saRNA variants, payload expression, and cytotoxicity on Huh7.5.1 cells. The results indicated that, at 24 h post‐transfection, the wt saRNA exhibited a significantly higher copy number of subgenomic RNA compared to the mutants harboring single mutations (Figure 2B). Similar results were also seen in Hela cells (Figure S2, Supporting Information). However, the replication of mutants containing two or more mutations was severely compromised.

#### 2.2.2 Reporter Gene Expression

Subsequently, we measured the levels of secreted nano luciferase expression from various mutants. Mutants 191 and 239 demonstrated significantly higher expression levels compared to the wt saRNA, while mutant 190 showed no significant difference. Consistent with their strongly impaired replication capacity, mutants carrying multiple mutations exhibited low expression levels (Figure 2C).

#### 2.2.3 Cytotoxicity

Lastly, we assessed the viability of cells transfected with mutant saRNAs. Despite a notable reduction in cell viability observed in all saRNA‐transfected cells, the three mutants exhibited significantly lower cytotoxicity compared to the wt saRNA across all tested time points (Figure 2D). The cell viability of mutant 191 slightly decreased compared to the other two mutant saRNAs after 5 days of transfection.

### 2.3 Amino Acid Mutation in the Macrodomain (MD) Impaired Mono ADP Ribose (MAR) Hydrolysis Activity and Replication Capability of saRNA

#### 2.3.1 Locations of Mutations in the saRNA

**Figure 3:**
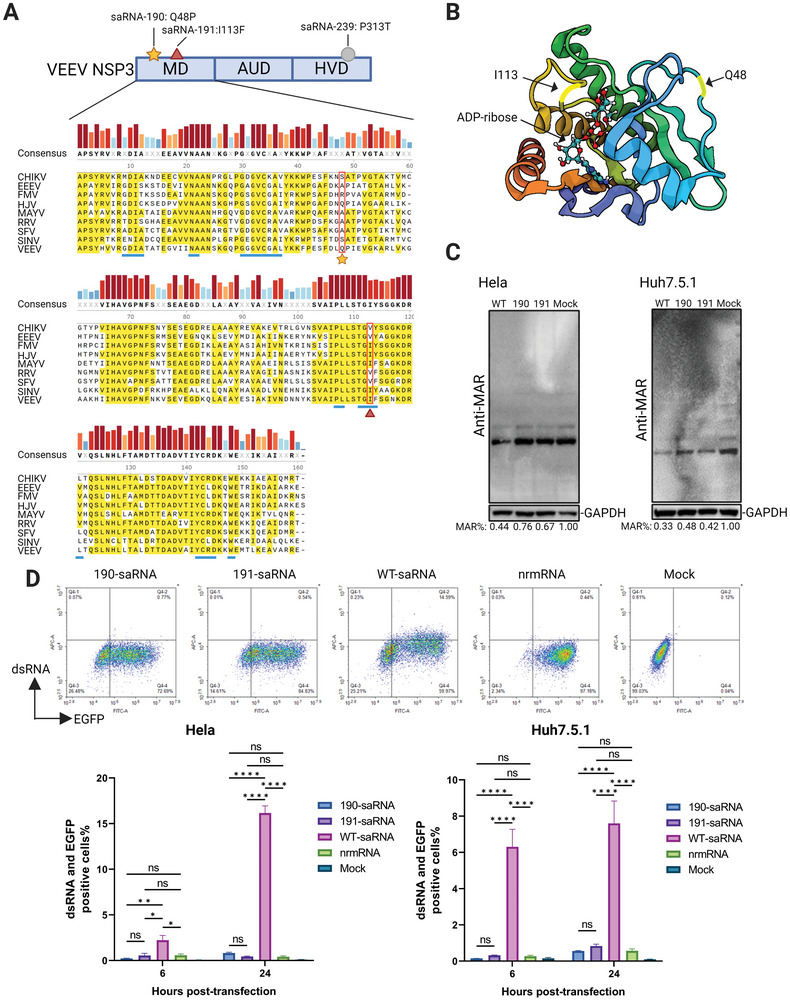
Functional determination of adaptive mutation in the macrodomain of saRNA. A) Position of identified adaptive mutation in saRNA, and amino acid sequence alignment of different viral macrodomains. MD represents the macrodomain, AUD stands for the alphavirus‐unique domain, and HVD for the hypervariable domain. Distinct mutations are denoted by unique shapes, the amino acids with homology exceeding 50% are marked in yellow in the alignment, and the amino acids in the ADPr binding pocket of VEEV MD are underlined in blue. CHIKV, Chikungunya virus; EEEV, Eastern equine encephalitis virus; FMV, Fort Morgan virus; HJV, Highlands J virus; MAYV, Mayaro virus; RRV, Ross River virus; SFV, Semliki Forest virus; SINV, Sindbis virus. B) Schematic of the mutation position in the VEEV MD. VEEV MD consists of four α‐helices and six β‐strands with narrow pocket binding and hydrolyzing ADPr. Bright yellow indicates the positions of mutation corresponding to 190 or 191. The arrow indicates the binding position of ADPr with the MD. C) In cell MAR hydrolysis activity of mutant saRNA. 24 h after the 190, 191, and wt saRNA transfected into Hela or Huh7.5.1 cells, the cellular MAR level was detected using immunoblot. The level of total protein MAR of different groups was normalized to GAPDH. D) Comparison of dsRNA formation in transfected cells: HeLa or Huh7.5.1 cells were transfected with saRNAs or nrmRNA, and dsRNA levels were assessed at 6 and 24 h post‐transfection using anti‐dsRNA antibodies, and flow cytometric analysis. An example of flow cytometry measurements showing dsRNA levels in HeLa cells at 24 h post‐transfection is presented. Statistical significance was determined using two‐way ANOVA with Tukey's multiple‐comparison test (^*^, *p* < 0.05; ^**^, *p* < 0.01; ^****^, *p* < 0.0001; ns, not significant).

Upon further analysis of the sequences, it was revealed that the mutations in positions 190 and 191 are both situated within the macrodomain (MD) of NSP3, whereas the mutation in position 239 is found in the NSP3 hypervariable domain (**Figure** **3**A). Considering the low homology and high susceptibility to insertions and deletions within the hypervariable domain among alphaviruses,^[^ ^27^ ^]^ we opted for a more conserved MD for subsequent investigation, taking into account the stability of the optimized saRNA.

Figure 3A also presents an alignment of amino acid sequences of the MD from various alphaviruses. The MD functions as an ADP‐ribose (ADPr) hydrolase expressed by positive‐sense RNA viruses such as coronaviruses, alphaviruses, and hepaciviruses,^[^ ^28^, ^29^, ^30^, ^31^ ^]^ with highly conserved amino acid sequences and structures among different viruses (Figure 3A,B). Sequence alignment with MDs from different viruses revealed that the mutation corresponding to position 190 is located at the 48th amino acid, which is less conserved across all MDs, whereas the mutation at position 191, occurring at the 113th amino acid, is significantly more conserved with a homology exceeding 50%. Structurally, I113 is precisely positioned at the ADPr binding pocket, facilitating direct interaction with ADPr. However, Q48 is situated on the side chain outside of the activity pocket.

#### 2.3.2 Reduced Levels of MAR Hydrolysis in Mutant 190 and 191

Due to the potential association of VEEV MD function with antiviral innate immunity, we broadened our investigation beyond RIG‐I‐deficient Huh7.5.1 cells to include RIG‐I‐normal HeLa cells. Using a monoclonal antibody that binds to mono ADP‐ribose, we conducted immunoblot analysis on MAR levels of total cellular proteins following saRNA transfection to assess MD function. Not surprisingly, transfection with wt saRNA led to a significant decrease in MAR levels compared to the Mock group at 24 h, whereas both mutant 190 and 191 exhibited much less reduction (Figure 3C). However, differences in the reduction of MAR levels among wt and mutants were less pronounced in Huh7.5.1 cells.

#### 2.3.3 Reduced dsRNA Level

Since replication of saRNA produces high amounts of dsRNA, which triggers strong innate immune responses in the transfected cells, we measured dsRNA levels in the transfected cells. Consistent with reduced replication, mutants 190 and 191 exhibited much lower dsRNA levels in both HeLa and Huh7.5.1 cells, showing levels similar to the nrmRNA group (Figure 3D; Figure S3, Supporting Information).

### 2.4 The Mutant saRNAs Induce a Less Intrinsic Innate Immune Response

**Figure 4:**
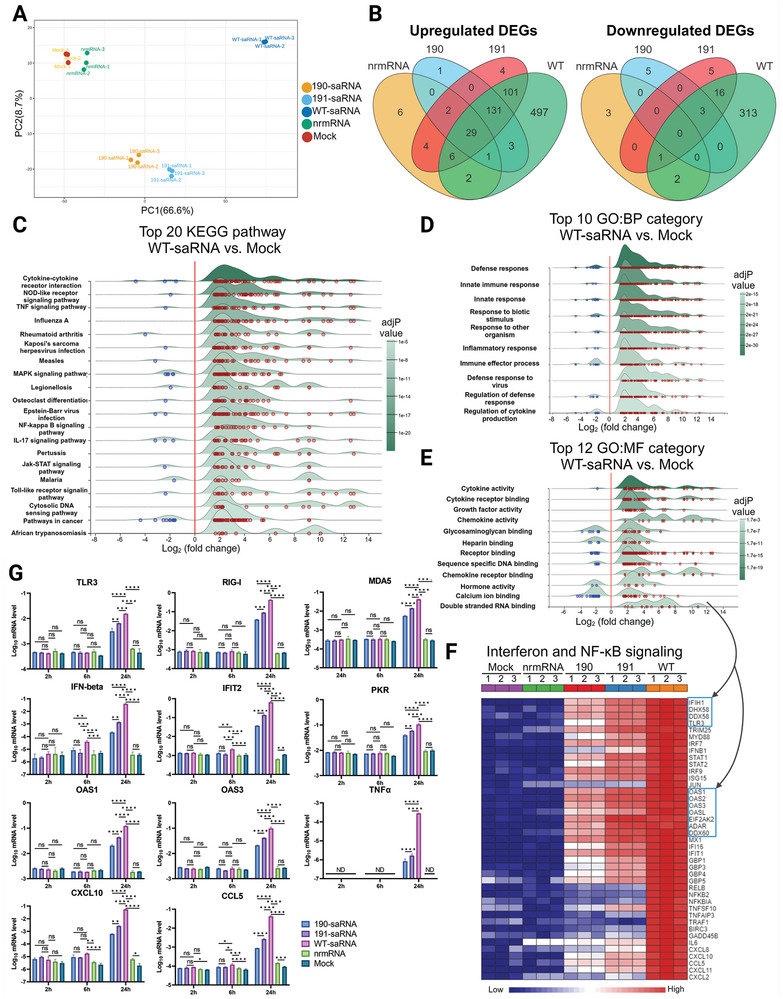
The innate immune response is induced by different saRNAs. A) Principal component analysis of transcriptome changes at 24 h post‐transfection with indicated RNAs in Hela cells. B) The Venn diagrams of the upregulated or downregulated gene numbers in each RNAs group versus the mock group. C) Ridgeline plot of KEGG and D) GO: biological process or E) GO: molecular function enrichment analysis for the differentially expressed gene in wt saRNA group versus the mock group. The panel respectively displays the top pathways or top categories enriched. Blue dots and red dots represent each downregulated or upregulated gene, and the x‐axis represents the fold change of indicated genes. The darkness of green indicates the adjusted P value of each pathway or category. F) Heatmap of the transcriptional difference in interferon and NF‐κB signaling pathway, the blue box indicated a set of upregulated dsRNA binding protein after transfected into Hela cells for 24 h. G) mRNA level changes of a key gene in innate immune response, the cellular RNA was extracted from HeLa cells at 2, 6, and 24 h post‐transfection with indicated RNAs for qPCR assay. Significance was determined by two‐way ANOVA with Tukey's multiple‐comparison test. ^*^, *p* < 0.05; ^**^, *p* < 0.01; ^***^, *p* < 0.001; ^****^, *p* < 0.0001. ns, no significance.

To gain insight into the changes in cellular activities following transfection with saRNA, we extracted total cellular RNA from HeLa cells transfected with mutant 190, 191, wt saRNAs, and also nrmRNA, respectively, for transcriptome analysis. Principal component analysis (PCA) revealed that compared to mock‐transfected cells, the nrmRNA induced minimal transcriptomic changes, while wt saRNA induced significant transcriptome disparity, far exceeding the changes observed in cells transfected with mutants 190 and 191 (**Figure** **4**A).

Furthermore, there were significant disparities in gene expression changes among cells transfected with nrmRNA, mutant saRNA, and wt saRNA. Generally, more genes were upregulated than downregulated across all groups (Figure S4, Supporting Information). Specifically, 770 genes were upregulated in wt saRNA‐transfected cells, compared to only 50 genes in the nrmRNA group. For the mutant saRNAs, 167 and 277 genes were upregulated in mutants 190 and 191, respectively. Among these upregulated genes, 76%, 98.2%, and 96.4% overlapped with those in the wt saRNA group for nrmRNA, mutant 190, and mutant 191, respectively. Conversely, 335 genes were downregulated in the wt saRNA group, while only 6, 8, and 25 genes were downregulated in the nrmRNA, mutant 190, and mutant 191 groups, respectively (Figure 4B). Some of these downregulated genes were also observed in the wt saRNA group (Table S1, Supporting Information).

Following this, we conducted kyoto encyclopedia of genes and genomes (KEGG) and gene ontology (GO) enrichment analysis on the differentially expressed genes between the wt saRNA group and the mock group. The results revealed that among the top 20 significantly different KEGG pathways or top 10 GO biological processes (BP) categories, most were related to immune responses, with the majority of genes being upregulated (Figure 4C,D). Particularly noteworthy was the upregulation of pathways associated with Influenza A, measles, and Epstein‐Barr virus infection, suggesting that wt saRNA triggered an innate immune response similar to viral infection.

In terms of specific pathways, the pattern recognition receptor pathways including NOD‐like receptor, and Toll‐like receptor signaling pathways were activated. Among these pathways, dsRNA recognition sensors such as TLR3, RIG‐I, MDA5, and DHX58 were strongly upregulated, subsequently inducing downstream transcription factors such as interferon regulatory factor 7 (IRF7) and IFN‐beta (Table S2, Supporting Information). Consequently, transcription factors such as STAT1, STAT2, and IRF9 in the Janus kinase‐signal transducer and activator of transcription (Jak‐STAT) pathway were significantly upregulated upon stimulation by IFN, resulting in the production of antiviral interferon‐stimulated genes (ISGs) such as OAS, eukaryotic translation initiation factor 2 alpha kinase 2 (EIF2AK2), adenosine deaminase acting on RNA (ADAR), MX dynamin‐like GTPase 1 (MX1), and IFIT. This indicates that dsRNA receptors play a key role in recognizing saRNA and initiating the interferon signaling pathway. Additionally, we observed activation of the NF‐κB signaling pathway. Upregulation of key transcription factors NF‐κB2 and RELB led to the induction of a large set of cytokines and chemokines including TNF, CXCL10, IL6, and CCL5, potentially initiating downstream TNF signaling pathways. Further analysis of the molecular functions (MF) of differentially expressed genes revealed that the functions of upregulated genes were related to cytokine, chemokine, growth factor, receptor binding, as well as double‐stranded RNA binding (Figure 4E). It's noteworthy that all upregulated dsRNA binding proteins are ISGs associated with direct antiviral function.

Moreover, the enrichment analysis of downregulated genes in the wt saRNA group indicated that, despite being enriched in specific pathways, there was no statistical significance except for calcium ion binding, and barely any association with the immune response was found (Tables S3,S4, Supporting Information).

For mutant saRNAs, the alteration of KEGG‐enriched pathways was nearly identical to those observed in the wt saRNA group (Figure S5, Supporting Information), suggesting that mutant saRNA shares the same pattern of induction of the innate immune response as wt saRNA. However, mutant saRNAs induced significantly fewer changes compared to wt saRNA. In contrast, the KEGG‐enriched pathways for nrmRNA did not show similar changes to those of saRNA, with only two pathways being significantly enriched. A gene heatmap comparing different RNA groups showed remarkable differences in levels of upregulated genes in the interferon and NF‐κB signaling pathways, indicating that wt saRNA elicited the strongest response, followed by mutant saRNAs, while nrmRNA was very close to the mock group, with barely any significant difference (Figure 4F).

Furthermore, we validated the mRNA levels of genes involved in the interferon and NF‐κB signaling pathways at various time points using qPCR (Figure 4G). Within the first 2 h, the mRNA levels in all groups remained unchanged compared to the mock transfection. Subsequently, the transcription of IFN, IFIT2, and CXCL10 increased at 6 h, indicating that activation of IRF7 and NF‐κB occurred between 2 and 6 h after transfection. At 24 h after transfection, all saRNA groups but not the nrmRNA group, showed significant induction of the tested genes. Again, the levels of upregulation of these genes followed the order: wt saRNA > mutant 191 saRNA > mutant 190 saRNA > nrmRNA ≈ mock.

### 2.5 Mutant saRNA Induces Less Translation Inhibition and Cell Apoptosis Leads to Enhanced In Vitro and In Vivo Payload Expression

#### 2.5.1 Mutant saRNA Showed Less Payload mRNA Copies but Higher Translational Efficiency

**Figure 5:**
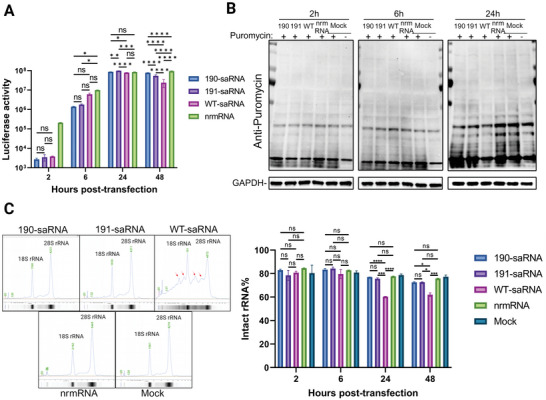
Assessment of translation inhibition induced by different saRNA. A) The expression levels of nano luciferase in the supernatant of HeLa cells at 2, 6, 24, and 48 h after transfection with indicated RNAs. B) Cellular translation activity at 2, 6, and 24 h post‐transfection with indicated RNAs. Before cell lysate collection, 10 µg mL^−1^ puromycin was added to the cell culture for 10 min to label the expressing protein, then the incorporated puromycin was detected by immunoblotting, and the GAPDH serves as internal control. C) Ribosomal RNA integrity was assessed in HeLa cells at 2, 6, 24, and 48 h post‐transfection with the indicated RNAs. An example illustrating ribosomal RNA integrity in HeLa cells at 24 h post‐transfection is provided, with a red arrow indicating degraded ribosomal RNA. Significance was determined by two‐way ANOVA with Tukey's multiple‐comparison test. ^*^, *p* < 0.05; ^***^, *p* < 0.001; ^****^, *p* < 0.0001. ns, no significance.

Given the variances observed in the elicited innate immune responses by distinct saRNAs, our investigation delved into the influence of mutations on payload mRNA transcription and translation. As depicted in Figure S2 (Supporting Information), the mRNA copy numbers of EGFP expressed by mutants were notably lower than those expressed by wt saRNA, indicating diminished transcription of the payload gene in mutants. We further assessed the protein expression of the payload by quantifying luciferase activity. The findings revealed that the payload expression from wt saRNA surpassed that of mutant saRNAs at 2 or 6 h post‐transfection into Hela cells. However, at the 24 h mark, the expression levels of wt saRNA were surpassed by mutants 191. Notably, by 48 h post‐transfection, mutant 190 exhibited nearly four times higher expression than wt saRNA (**Figure** **5**A), while wt saRNA expression decreased. For nrmRNA, its expression level continued to increase throughout the period and remained higher than those of the saRNA groups. Considering that the mutants showed lower transcriptional activity but higher protein levels, we hypothesize that the translation of the payload in mutant 190 saRNA must be more efficient than in wt saRNA.

#### 2.5.2 Payload Translation was Inhibited in wt saRNA

Figure 5B presents the protein translation activity of cells following saRNA transfection, assessed via immunoblotting to measure the incorporation of puromycin into translating polypeptides. Interestingly, at 2 and 6 h post‐transfection, no significant difference in protein translation efficiency was observed among all groups. However, at 24 h post‐transfection, the translation activity of wt saRNA was markedly lower than that of mutant saRNAs, while nrmRNA exhibited comparable translation activity to the mock group.

In Figure 4F, we observed significant upregulation of dsRNA‐binding proteins OAS1‐3 following saRNA transfection. OASs are pivotal in the OAS‐RNase L pathway, which regulates the stability of ribosomal RNA (rRNA), thereby impacting translation efficiency. Consequently, we speculate that protein translation of wt saRNA is hindered when OASs are substantially upregulated.

To explore this, we assessed the integrity of rRNA at various time points post‐saRNA transfection (Figure 5C; Figure S6, Supporting Information). Our results revealed that cells transfected with wt saRNA exhibited relatively intact rRNA at 2 and 6 h, but substantial degradation occurred at 24 and 48 h. In contrast, the integrity of rRNA in cells transfected with mutants 190, 191, and nrmRNA showed no significant decrease compared to the mock group. This suggests that rRNA degradation occurs subsequent to wt saRNA transfection, likely as a consequence of the activation of the OAS‐RNase L pathway.

#### 2.5.3 Mutant saRNAs Induced Less Apoptosis

**Figure 6:**
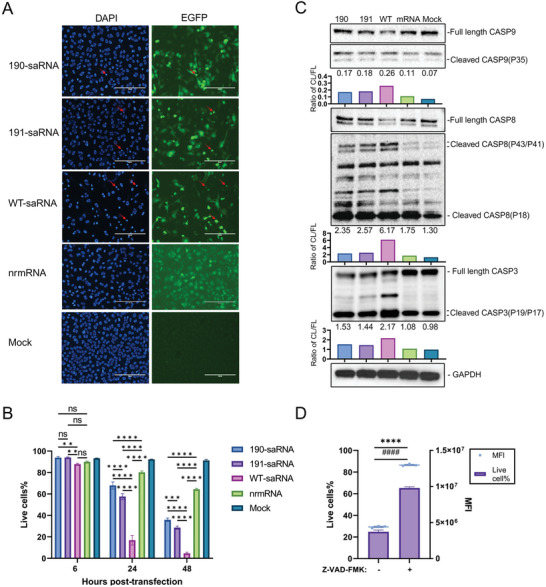
Assessment of cell apoptosis induced by different saRNA. A) Fluorescence images showing nuclei morphology and EGFP expression in HeLa cells transfected with different RNAs at 24 h. The red arrows indicate condensed nuclei in RNA‐transfected cells. B) The proportion of non‐apoptotic live cells was assessed at 6, 24, and 48 h post‐transfection with indicated RNAs, using Annexin V and PI staining. Statistical significance was determined by two‐way ANOVA with Tukey's multiple‐comparison test (^**^, *p* < 0.01; ^***^, *p* < 0.001; ^****^, *p* < 0.0001; ns, not significant). C) Activation of caspases 8, 9, and 3 in HeLa cells was assessed by western blot assay after transfection with indicated RNAs for 24 h. D) The proportion of non‐apoptotic live cells and EGFP expression intensity in wt saRNA transfected Hela cells after treatment with 20 µM pan‐caspase inhibitor Z‐VAD‐FMK. Significance was determined by a two‐tailed unpaired t‐test. ^****^ or ^####^, *p* < 0.001.

At 24 h post‐transfection with wt saRNA, we observed significant nuclear condensation, indicative of apoptosis in the cells (**Figure** **6**A). To validate this observation, annexin‐V, and PI staining were performed on cells transfected with different saRNAs for 24 and 48 h. As depicted in Figure 6B and Figure S7 (Supporting Information), all RNAs induced apoptosis post‐transfection compared to the mock transfection. Although cell death induced by mutants 190 and 191 was greater than that induced by nrmRNA, it was notably less pronounced than that induced by wt saRNA.

Furthermore, western blot analysis of the apoptosis initiator caspase 8 and caspase 9, along with effector caspase 3, revealed significant activation of these caspases in cells transfected with saRNAs compared to the nrmRNA or mock group. Once again, the activation of caspases in the wt saRNA group was considerably more pronounced than in the mutants (Figure 6C). These findings suggest that saRNAs activate both caspase 8‐mediated extrinsic and caspase 9‐mediated intrinsic apoptosis pathways.

Additionally, treatment with a pan‐caspase inhibitor in cells transfected with wt saRNA effectively inhibited apoptosis and enhanced the mean fluorescence intensity of expressed EGFP threefold (Figure 6D), indicating that apoptosis affects payload expression efficiency.

#### 2.5.4 Mutant saRNA had a Higher Level Expression of the Reporter Gene In Vivo

**Figure 7:**
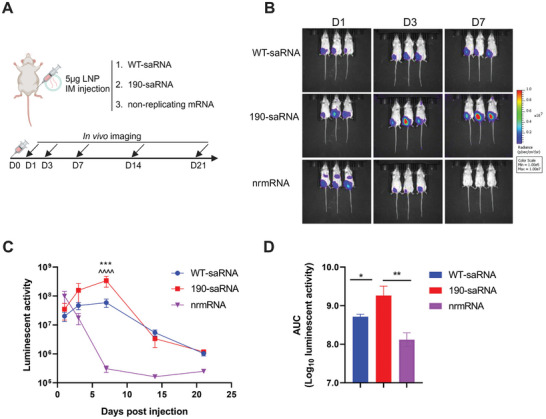
Comparison of in vivo expression level of LNP encapsulated 190, wt saRNA or nrmRNA. A) The schematic of RNA‐LNP immunization and detection. 4–6 weeks old Balb/c mice (N = 3/group) were intramuscularly immunized with 5 µg of RNA‐LNP expressing firefly luciferase for in vivo imaging. B) In vivo expression images on days 1, 3, and 7 post‐injections. C) The time course curve of RNA‐LNP expression level. Significance was determined by two‐way ANOVA with Tukey's multiple‐comparison test. ^***^, *p* < 0.001 (190 versus WT). ^^^^^^, *p* < 0.0001 (190 versus nrmRNA). D) The area under the curve results in the in vivo expression levels. Significance was determined by a one‐tailed unpaired t‐test. ^*^, *p* < 0.5; ^**^, *p* < 0.01.

To investigate whether mutations cause any differences in saRNA expression levels in vivo, we encapsulated 190 saRNA, wt saRNA, and nrmRNA expressing firefly luciferase in lipid nanoparticles (LNPs) for intramuscular injection of mice, followed by subsequent in vivo imaging (**Figure** **7**A). The results revealed that the expression levels of nrmRNA were slightly higher than saRNA on the first day after injection but declined quickly thereafter, becoming almost undetectable after 7 days (Figure 7B,C). In contrast, for saRNA, 190 exhibited a continuous increase in expression levels within 7 days, surpassing wt saRNA. Although it maintained the same level as wt saRNA after 14 days, the accumulated expression level remained higher for 190 compared to both wt saRNA and nrmRNA throughout all time points (Figure 7D).

## 3 Conclusion

High‐level and durable payload expression of saRNA is the outcome of continuous amplification of subgenomic RNA, it is reasonable to deduce that the enhancement of saRNA replication capacity will increase the overall payload expression. However, our findings provide a new perspective, as the replication intermediate dsRNA may trigger the host's innate response, resulting in increased rRNA degradation, reduced protein synthesis, and apoptotic cell death. Here, utilizing in vitro evolution we screened a few adaptive mutations located in the MD that attenuated the replication capacity of saRNA, causing a subdued innate immunity. Consequently, the mutant saRNAs demonstrate enhanced protein expression and reduced cytotoxicity both in vitro and in vivo.

Acquiring adaptive mutations through in vitro directed evolution is one of the strategies to improve saRNA expression potency, Petrakova et al. employed serial passaging of a VEEV‐derived replicon expressing PAC on interferon‐deficient BHK‐21 cells, acquired a noncytopathic adaptive mutations located in NSP2, enhancing saRNA expression levels.^[^ ^32^ ^]^ In a similar vein, Li et al. screened several replicase mutations that enhanced gene expression by subjecting saRNA to in vitro evolution coupled with flow cytometry‐based cell sorting in interferon‐competent Jurkat cells.^[^ ^33^ ^]^ Additionally, a study on protein evolution based on saRNA platform, known as Viral Evolution of Genetically Actuating Sequences (VEGAS), demonstrated that by applying appropriate external pressures, the transcription factor activity encoded by saRNA could be elevated by an order of magnitude within a week of evolution,^[^ ^34^ ^]^ highlighting the efficiency of saRNA‐based directed evolution. In our study, we selected Huh7.5.1 cells that are capable of supporting stable saRNA replication in the presence of low concentrations of IFN‐alpha. Two saRNAs carried MD mutation remained active with persistent protein expression of their encoded reporter gene during the serial passaging despite exogenous IFN‐alpha pressure. Consistent with our findings, several studies reported that the decline in binding or hydrolytic activity of the viral MD leads to replication attenuation. MD mutations, such as G32S and Y114 in the Chikungunya virus or N10A in the Sindbis virus, resulted in reduced ADPr‐binding and hydrolase activities, leading to less efficient replication,^[^ ^31^, ^35^ ^]^ demonstrating the structural and functional conservation of the viral MD.

The innate immune response to alphavirus is initiated when RNA sensors recognize the RNA pathogen associated molecular patterns, thus RNA sensors hold the first line against alphavirus infection. It is known that RIG‐I and MDA5 are dsRNA sensors essential in sensing alphavirus replication and initiating innate immune responses via IFN or NF‐κB signaling.^[^ ^36^ ^]^ By competitively binding to MDA5 using the 5 V protein of parainfluenza virus or M protein of Middle East respiratory syndrome coronavirus,^[^ ^20^ ^]^ the saRNA‐induced antiviral response was inhibited and payload expression enhanced, highlighting the surveillance function of RNA sensors in innate response.

Our results confirmed that the dsRNA sensors TLR3, RIG‐I, and MDA5 are substantially upregulated by saRNA transfection at 24 h compared to nrmRNA. However, in addition to the dsRNA sensors, other innate immune response genes (IFN‐beta, IFIT2, CXCL10, and CCL5) were also upregulated as early as 6 h post‐transfection for wt saRNA but not for the mutants (Figure 4G). Therefore, reduced dsRNA levels may not be the only mechanism for the attenuation of the innate immune response by mutations in the MD of saRNA. The mutants may also delay the onset of other innate immune responses, contributing to the reduced immune response in the transfected cells. Nevertheless, rRNA degradation and reduced protein synthesis occurred at 24 h post‐transfection, coinciding with the activation of dsRNA sensors, suggesting that dsRNA sensor‐induced innate immunity may still be the primary cause of reduced payload expression.

Upon recognition of PAMPs by the RNA sensors, a common set of transcription factors like IRF3, IRF7, and NF‐kB are activated to induce type I IFN and expression of inflammatory cytokines, followed by activation of ISGs in IFN‐JAK‐STAT axis including PKR and OAS.^[^ ^37^ ^]^ The two are responsible for translation shutoff and OAS/RNase L‐mediated rRNA degradation.^[^ ^38^ ^]^ Meanwhile, the apoptotic pathway is also activated through both intrinsic and extrinsic pathways after alphavirus infection to restrict replication and expression.^[^ ^39^, ^40^ ^]^ saRNA‐induced apoptosis triggered by TLR3 activation has also been reported in B16F10 cells.^[^ ^6^ ^]^ We found that the caspase 8/9‐dependent apoptosis also activated in Hela cells, suggesting multiple apoptosis mechanisms involved, such as extrinsic apoptosis through TLR3 signaling,^[^ ^41^, ^42^ ^]^ TNFα‐mediated death receptors signaling.^[^ ^43^ ^]^ Additionally, the OAS‐RNase L pathways may also lead to apoptosis after dsRNA stimulation.^[^ ^44^, ^45^ ^]^ Although our experiment utilizing a pan‐caspase inhibitor successfully rescued over 50% of saRNA‐transfected cells, indicating an apoptotic mode of cell death, we must acknowledge the potential involvement of pyroptosis and necroptosis mechanisms. Recent discoveries of dsRNA sensors such as NLRP1, NLRP6, and ZBP1 suggest their potential roles in initiating these alternative pathways of cell demise.^[^ ^46^, ^47^, ^48^, ^49^ ^]^

Taken together, our current study indicates that, unlike conventional mRNAs, a strong innate immune response to saRNA occurs at later stages due to RNA amplification. While initial single‐stranded saRNA may provoke an early innate immune response, the intermediate dsRNA formed during RNA‐dependent RNA replication is a crucial factor influencing the innate immune response and payload expression. Our results showed that while saRNA is superior to nrmRNA in payload expression in vivo, the substantial innate immune response induced by wt saRNA can be a disadvantage. The strong innate immune response triggered by wt saRNA may not pose a major safety concern, as demonstrated by the recently approved COVID‐19 vaccine ARCT‐154, which has shown good clinical tolerability.^[^ ^50^ ^]^ However, our findings indicate that the overactivated innate immune response induced by wt saRNA can result in reduced payload expression. Therefore, it is important to adequately manage the balance between the copy number of dsRNA and the innate immune response to achieve high levels and prolonged protein expression by saRNAs. Nevertheless, whether the reduced innate immune response observed with mutant saRNAs at the cellular level is the sole reason for increased protein expression still needs to be further validated in more complex in vivo environments. Similarly, the potential impact of the cytotoxicity induced by saRNAs on in vivo or clinical applications also needs to be carefully investigated in future studies. Overall, our results demonstrate that by modifying the alphavirus components of the saRNA backbone, it is possible to generate saRNA variants with reduced adverse effects of innate immune response to produce higher levels of payload expression.

## 4 Experimental Section

### Cell Culture and RNA Transfection

The human cervical cancer cells HeLa, Baby Hamster Kidney‐21 cells (BHK‐21), immortal murine macrophage RAW264.7, murine myoblast cells C2C12 and Human hepatoma 7.5.1 (Huh7.5.1) cells were procured from the American Tissue Culture Collection (Manassas, Virginia, USA). The cells were cultured in Dulbecco's Modified Eagle medium (DMEM) supplemented with 10% fetal bovine serum (FBS, Gibco) and 1% penicillin and streptomycin (Gibco), for Huh7.5.1 cells, the medium was also supplemented with 1X MEM Non‐Essential Amino Acids Solution (MEM NEAA, Gibco), all cells were cultured at 37 °C and 5% CO_2_. The RNAs were transfected into cells using the TransIT‐mRNA Transfection Kit (Mirus), following the manufacturer's instructions. For serial passaging of saRNA, Huh7.5.1 cells were treated with 1pg mL^−1^, 1 ng mL^−1^, or 1 µg mL^−1^ recombinant human interferon alpha 2 protein (IFNα2, Sino Biological) for 3 h before transfection, the 4ug mL^−1^ Puromycin Dihydrochloride (Beyotime) was added to the medium at 24 h post‐transfection. The stable transfected Huh7.5.1 cells were maintained in the medium supplemented with 4ug mL^−1^ Puromycin Dihydrochloride and 1pg mL^−1^ IFNα2 and subculture for 14 passages, the saRNA stable‐transfected cells of 3, 7, and 14 passages were collected for mutation identification.

### Plasmid Construction

The saRNA was constructed by substituting the structure protein sequence of Venezuelan equine encephalitis virus (TC‐83 strain, GenBank: L01443.1) with porcine teschovirus 2A self‐cleaved peptide linked nano luciferase and EGFP fusion protein, or firefly luciferase encoding gene. For the nrmRNA, the encoded firefly luciferase or nano luciferase and EGFP fusion protein were flanked by 5′ and 3′ UTR of the Xenopus β‐globin gene, as described before.^[^ ^51^ ^]^ The saRNA or mRNA elements were flanked by T7 polymerase promoter at 5′ end and poly(A) tail plus BspQI cleavage site at 3′ end, all sequences were synthesized and cloned into a pUC vector by Azenta Life Science. For the saRNA used for serial passaging, the puromycin N‐acetyl‐transferase gene under the control of a second subgenomic promoter was introduced between the reporter gene and 3′ UTR. The adaptive mutation was introduced by in‐fusion cloning using ClonExpress Ultra One Step Cloning Kit (Vazyme).

### In Vitro Transcription

RNAs were prepared by Fraserna Life Sciences Limited. Briefly, all linearized plasmid templates for in vitro transcription of RNA were prepared by the digestion with BspQ I (Vazyme), then the templates were mixed with T7 RNA polymerase (Vazyme), nucleoside triphosphates (dNTPs) (Vazyme), Cap 1 analogs (Synthgene), RNase inhibitors (Vazyme) and inorganic pyrophosphatase (Vazyme), and incubate for 2 h at 37 °C. Then the Cap 1 analog capped and N1‐methylpseudouridine modified nrmRNA or Cap 1 analog capped but nucleotide unmodified saRNAs were purified by lithium chloride precipitation and dissolved in RNase‐free water. The integrity of the RNA was assessed by capillary electrophoresis using Qsep400 high‐throughput nucleic acid protein analysis system (Bioptic).

### Adaptive Mutation Identification and Quantitative Real‐Time PCR

The total RNA was extracted using FastPure Cell/Tissue Total RNA Isolation Kit (Vazyme) according to instruction, and the first‐strand cDNA was synthesized by reverse transcription with primer 198 using HiScript III 1st Strand cDNA Synthesis Kit (Vazyme). Then the saRNA NSP gene was amplified by PCR using primer 198 and 247, then constructed into the vector using Zero TOPO‐Blunt Cloning Kit (Sangon Biotech) subject to Sanger sequencing, at least ten clones of each passage were picked for mutation identification. For quantitative PCR assay, the first‐strand cDNA was synthesized using random hexamer primers and Oligo (dT)20VN primers (Vazyme), then qPCR was performed using SYBR Green Quantitative PCR Kit (Vazyme), cycling conditions were 95 °C for 30 s, and then 40 cycles of 95 °C for 10 s followed by 60 °C for 30 s with a melt curve of 95 °C for 15 s, 60 °C for 1 min, 95 °C for 15 s. The PCR was carried out using a QuantStudio5 Real‐Time PCR system (Thermofisher), the delta CT value was calculated by deducting the CT value of GAPDH by gene of interest, and the delta CT> 20 would be considered as negative, the 2^ (‐delta CT value) was calculated as mRNA level. The primers used were summarized in Table S5 (Supporting Information).

### In Vitro Luciferase Assay

The nano luciferase activity was measured using the Nano‐Glo Luciferase Assay Kit (Promega). Briefly, the collected supernatants collected at the indicated time point were transferred to a 96‐well opaque plate containing Nano‐Glo luciferase assay reagent in an equal volume to the supernatant, and luminescence intensity was measured after incubated at room temperature for 3 min.

### In Vitro Cytotoxicity Assay

Cytotoxicity was assessed using the Cell Counting Kit‐8 (Beyotime). In brief, CCK‐8 reagents were added to each well of the 96‐well plate at the indicated time point, then the absorbance was measured at a wavelength of 450 nm after incubation for 1 h, and the cell viability was normalized to the mock transfection group.

### Immunoblotting

Cells were lysed in Radio‐Immunoprecipitation Assay Lysis buffer (RIPA, Beyotime) containing 1 mm phenylmethanesulfonyl fluoride (PMSF, Beyotime) and Phosphatase Inhibitor Cocktail II(MCE). For puromycin detection, a culture medium containing 10ug mL^−1^ Puromycin Dihydrochloride (Beyotime) was substituted and incubated for 10 min before harvesting cells. After centrifuging for 12,000 rpm at 4 °C for 10 min, the supernatants were mixed 3:1 with 4X NuPAGETM LDS sample buffer (Invitrogen) and heated at 70 °C for 10mins, then separated by Bis‐Tris PAGE and transferred to polyvinylidene difluoride (PVDF) membranes, the blots were blocked with 5% nonfat milk and probed with the following antibodies, anti‐mono‐ ADP‐ribose binding reagent (Merck, MABE1076, 1:1000), anti‐puromycin(Abclonal, A21205, 1:1000), anti‐caspase‐3(Beyotime, AC030, 1:1000), anti‐cleaved caspase‐3(Beyotime, AC033, 1:1000), anti‐caspase‐8(CST, #4790, 1:1000), anti‐cleaved caspase‐8(CST, #9496, 1:1000), anti‐caspase‐9(Beyotime, AC062, 1:1000), anti‐GAPDH(Beyotime, AF1186, 1:1000). HRP‐conjugated Goat Anti‐Rabbit IgG H&L (Abcam, ab205718, 1:10000) or HRP‐conjugated Goat Anti‐Mouse IgG (Sangon, D110087, 1:10000) were used as secondary antibody, and the blots were visualized using BeyoECL Moon (Beyotime).

### Apoptosis Analysis

The Annexin V‐APC/PI apoptosis kit (MultiSciences) was used to evaluate apoptosis. For the caspase inhibition experiment, cells were pre‐treated with a 20um pan‐caspase Inhibitor Z‐VAD‐FMK (Beyotime) for 30 mins before transfection. Cells were detached by accutase enzyme, collected, washed twice with PBS, and resuspended in binding buffer. Subsequently, Annexin V‐APC solution and PI solution were added to all samples and incubated in the dark at room temperature for 5 min to be analyzed. At least 10000 events of EGFP‐positive cells were counted per sample for flow cytometry analysis.

### Double‐Stranded RNA Detection

The intracellular dsRNA was detected as described before.^[^ ^5^ ^]^ In short, the mouse monoclonal anti‐dsRNA antibody (IgG2a subtype, J2 clone, SCICONS) was labeled with Alexa Fluor 594 dye using Zenon Mouse IgG2a Labeling Kits (Invitrogen), next the cells were collected, fixed, and permeabilized before staining, then the dsRNA positive cells were measured by flow cytometry using ACEA NovoCyte (Agilent). At least 10000 events of EGFP‐positive cells were counted per sample for flow cytometry analysis.

### Bulk RNA Sequencing and Bioinformatic Analysis

The RNA sequencing was carried out by Azenta Life Science. First, the poly(A) mRNA was isolated from total RNA and fragmented. Then, the cDNA libraries were prepared using the commercial kits. Last, 2 × 150 bp paired‐end sequencing was conducted on an Illumina HiSeq instrument according to the manufacturer's instructions. The raw data were processed and confirmed by Cutadapt (V1.9.1), then aligned to the human genome (HG38) via Hisat2 (v2.2.1), and read counts of each transcript were estimated using HTSeq (v0.6.1). The differential expression analysis, principal component analysis, KEGG and GO enrichment analysis were conducted using ExpressAnalyst,^[^ ^52^, ^53^ ^]^ parameter of read count ≥ 10, adjusted *p* value ≤ 0.05 and log_2_ fold change ≥ 1.5 were set to detect the differentially expressed gene, and terms with adjusted *p* < 0.001 were considered significant.

### Ribosome RNA Integrity Assessment

The total RNA of saRNA transfected cells was extracted by the method described above, and then the equal amount of total RNA of each sample was analyzed using Qsep400 high‐throughput nucleic acid protein analysis system (Bioptic). The integrity of ribosome RNA was calculated according to Equation 1:

(1) $$\begin{array}{ccc} \mathrm{rRNA}\;\mathrm{integrity}\left(\%\right) & & =\frac{\mathrm{dist}\mathrm{ribu}\mathrm{tion}\;\mathrm{prop}\mathrm{orti}\mathrm{on}\;\mathrm{of}\;28S\;\mathrm{rRNA}\;\mathrm{and}\;18S\;\mathrm{rRNA}}{\mathrm{dist}\mathrm{ribu}\mathrm{tion}\;\mathrm{prop}\mathrm{orti}\mathrm{on}\;\mathrm{of}\;\mathrm{total}\;\mathrm{rRNA}}\\ & & *100\% \end{array}$$

### Lipid Nanoparticles Encapsulation

RNAs were dissolved in citrate buffer (pH 4.0, 50 mm) at a concentration of 0.2 mg mL^−1^. Four‐component lipids were dissolved in ethanol with molar ratios of 47.5:1.8:10.0:40.7 (ALC‐0315(DHA): ALC‐0159: DSPC: Cholesterol). The lipid and saRNA solutions were rapidly mixed using a commercial microfluidic mixer (Precision NanoSystems Inc, Cytiva, USA). The raw RNA‐LNPs solution was ultrafiltered in 100 kDa ultra‐centrifugal filters (Thermo Fisher Scientific) against 1 × DPBS (pH 7.4) buffer. After buffer exchange, 1.2 m sucrose in RNase‐free water was added to the mRNA‐LNPs solution as a cryo‐protectant. The final solution was sterilized using a 0.22 µm filter and stored at −80 °C.

### In Vivo Bioluminescence Imaging

The 6‐week‐old Balb/c female mice were intramuscularly injected at behind limb with 5 µg firefly luciferase encoding RNA‐LNP, and the in vivo imaging was performed at 1, 3, 7, 14, and 21 days after injection. 15 min before imaging, the mice were anesthetized with isoflurane and subcutaneously injected with 100 mg k^−1^g^−1^ luciferin, and then the luminescence of the injection site was measured using Lumina XRMS(PerkinElmer). All animal studies were approved by the Animal Care Committee of Shanghai Virogin Biotech Co., Ltd. (permission numbers: RD‐VC‐2023043).

### Software and Statistical Analysis

All experiments were performed at least twice in triplicates and data are presented as mean values ± standard error of the mean (SEM). ANOVA and student's t‐test comparison were performed using GraphPad Prism version 9.5.0. Amino acid sequence alignment was performed using MUSCLE version 3.8.1551. The band relative intensity of immunoblotting was quantified using ImageJ version 1.54 g. All figures were created with BioRender.com.

## Conflict of Interest

All authors are current employees of Virogin Biotech Co. Ltd., and the patent application related to this study is currently pending.

## Supporting information



Supporting Information

## Data Availability

The data that support the findings of this study are available from the corresponding author upon reasonable request.
