# Supplementary material for: A Novel Self‐Amplifying mRNA with Decreased Cytotoxicity and Enhanced Protein Expression by Macrodomain Mutations
Source: Adv Sci (Weinh). 2024 Sep 23;11(43):2402936. doi: 10.1002/advs.202402936 (PMC11578319; doi:10.1002/advs.202402936)
Supplement: Supplementary file 1 — Supporting Information [file ADVS-11-2402936-s001.docx]

Supporting Information

A novel self-amplifying mRNA with decreased cytotoxicity and enhanced protein expression by macrodomain mutations

Yue Gong, Danni Yong, Gensheng Liu, Jiang Xu, Jun Ding, and William Jia *


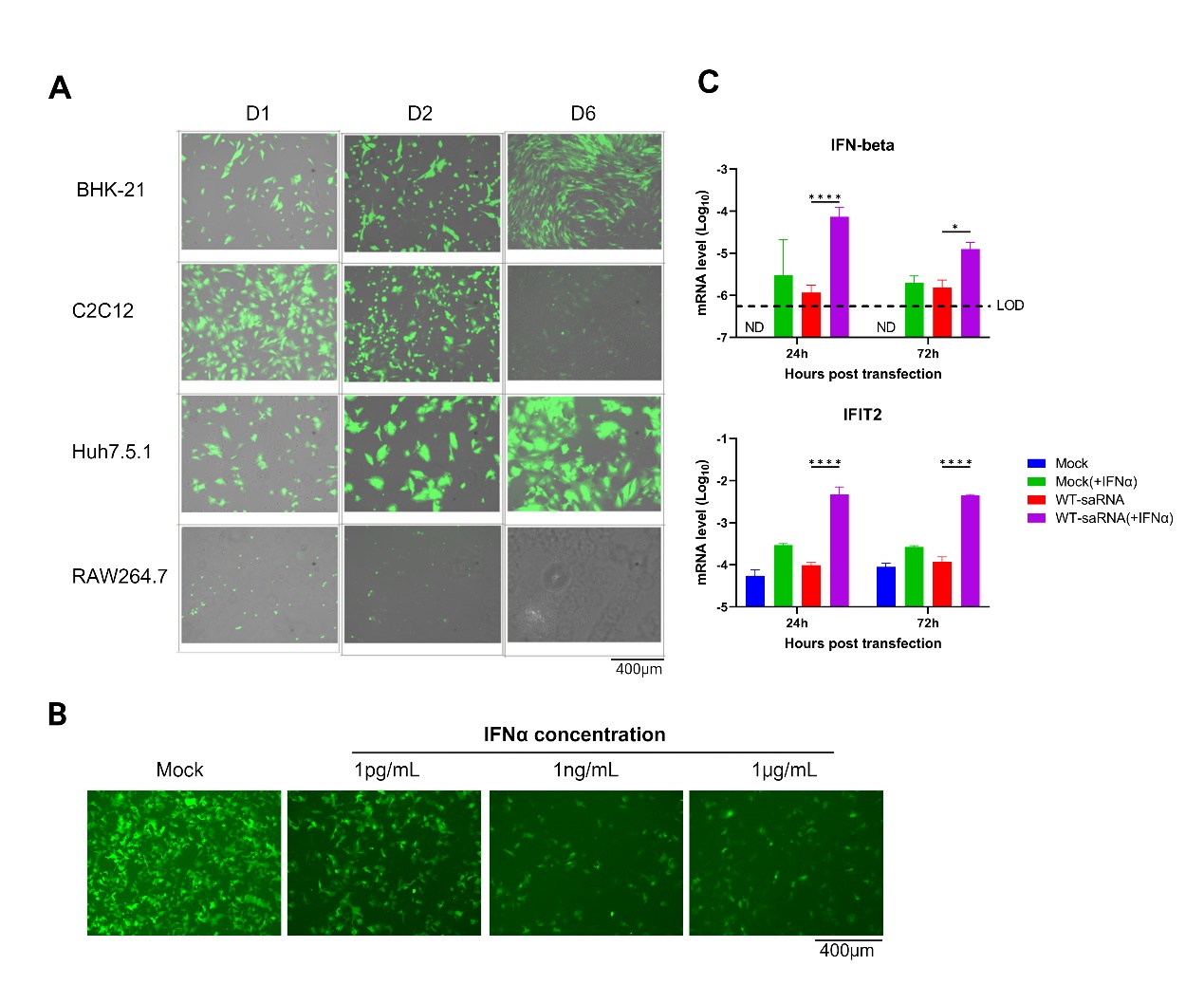


**Supplementary Figure 1**. Validation the interferon sensitivity of saRNA in various cells. A) Fluorescence images of EGFP expression in BHK-21, C2C12, Huh7.5.1, and Raw264.7 Cells at 1, 2, and 6 days after saRNA transfection. B) Fluorescence images of EGFP expression at 24 hours after saRNA transfection in Huh7.5.1 cells pre-treated with different concentrations of IFNα. C) Changes in IFN beta and IFIT2 mRNA levels in saRNA-transfected Huh7.5.1 cells with or without 1 pg/mL IFNα pretreatment. Significance was determined by two-way ANOVA with Tukey's multiple-comparison test. *, P < 0.05; ****, P < 0.0001. ND, not detected. LOD, limit of detection.


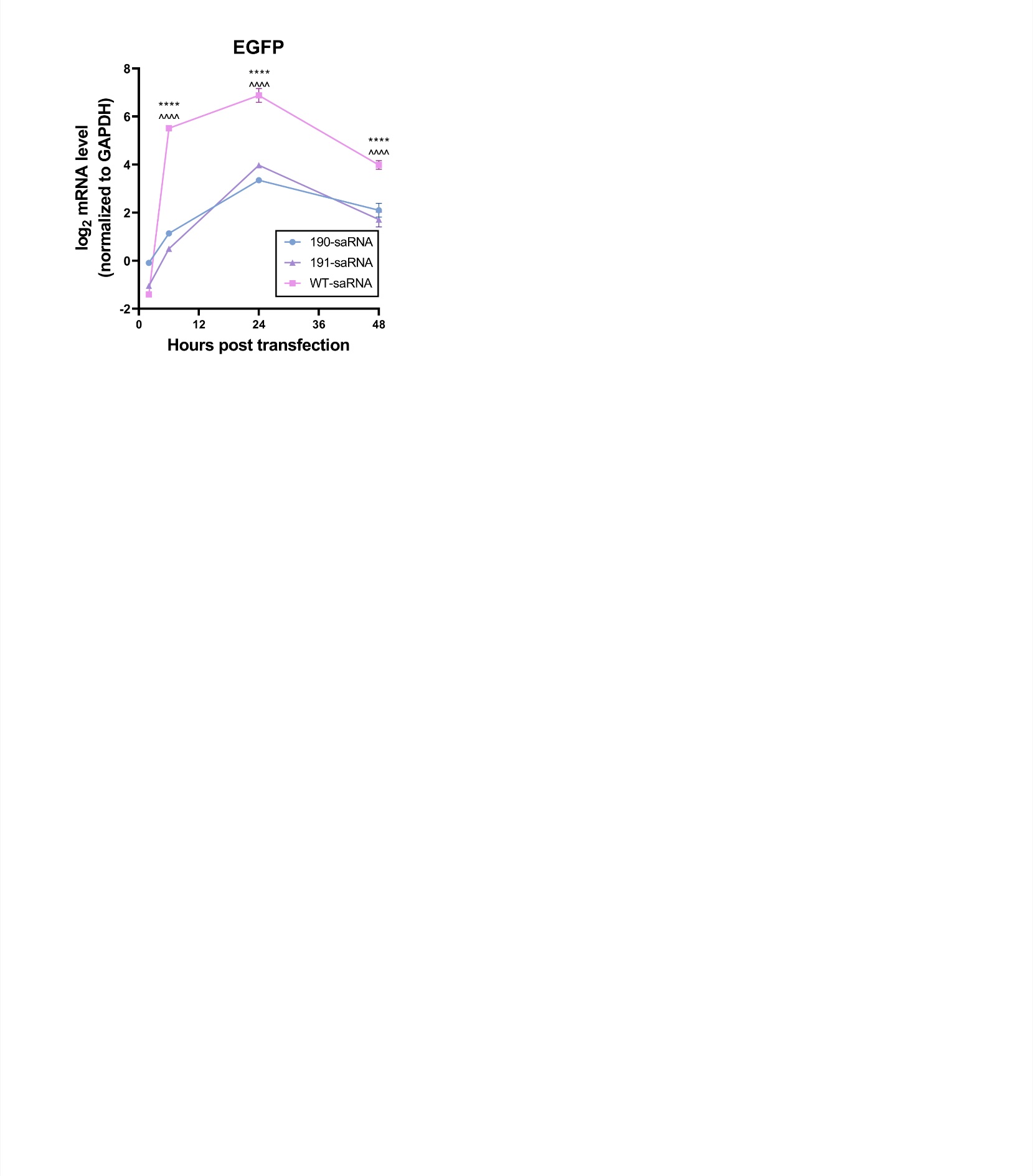


**Supplementary Figure 2.** Comparison of subgenomic RNA level in HeLa cells at 2, 6, 24, and 48 hours post-transfection of indicated saRNA. Significance was determined by two-way ANOVA with Tukey's multiple-comparison test. ****, P < 0.0001 (WT versus 190). ^^^^, P < 0.0001 (WT versus 191).


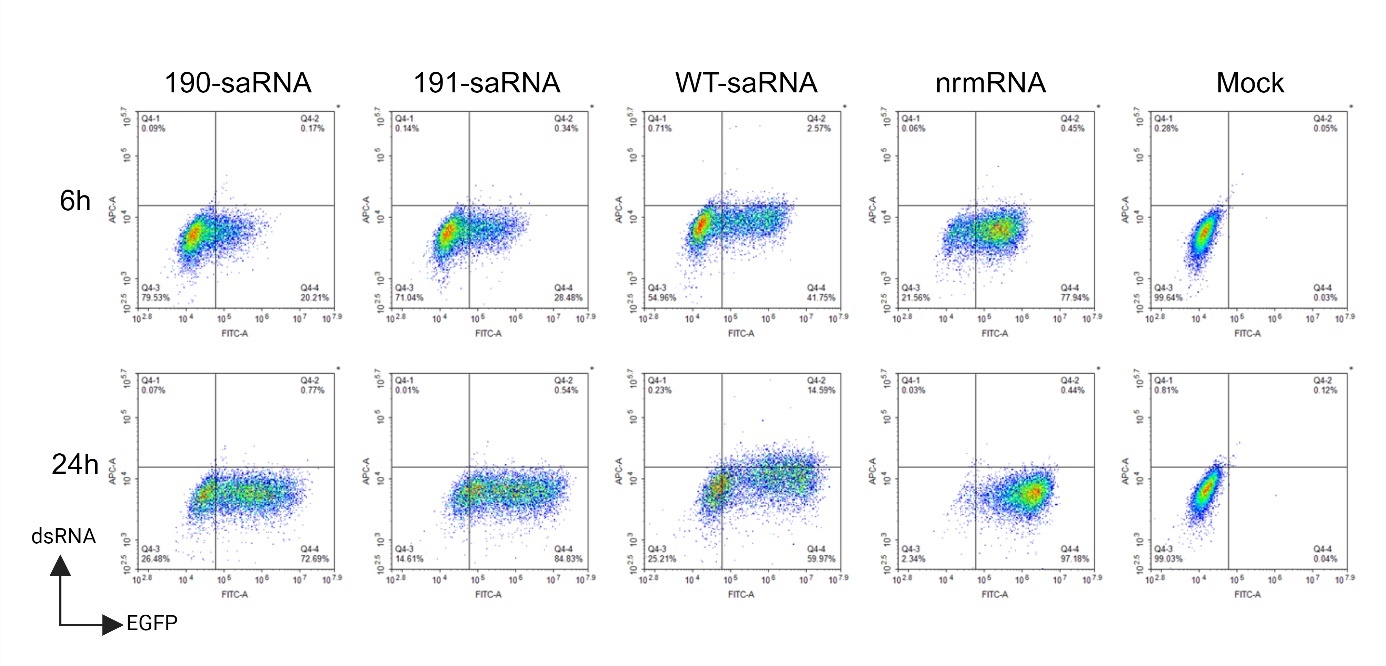


**Supplementary Figure 3.** The dsRNA formation analysis in HeLa cells at 6 and 24 hours after transfection with indicated RNAs. The mock group served as negative control.


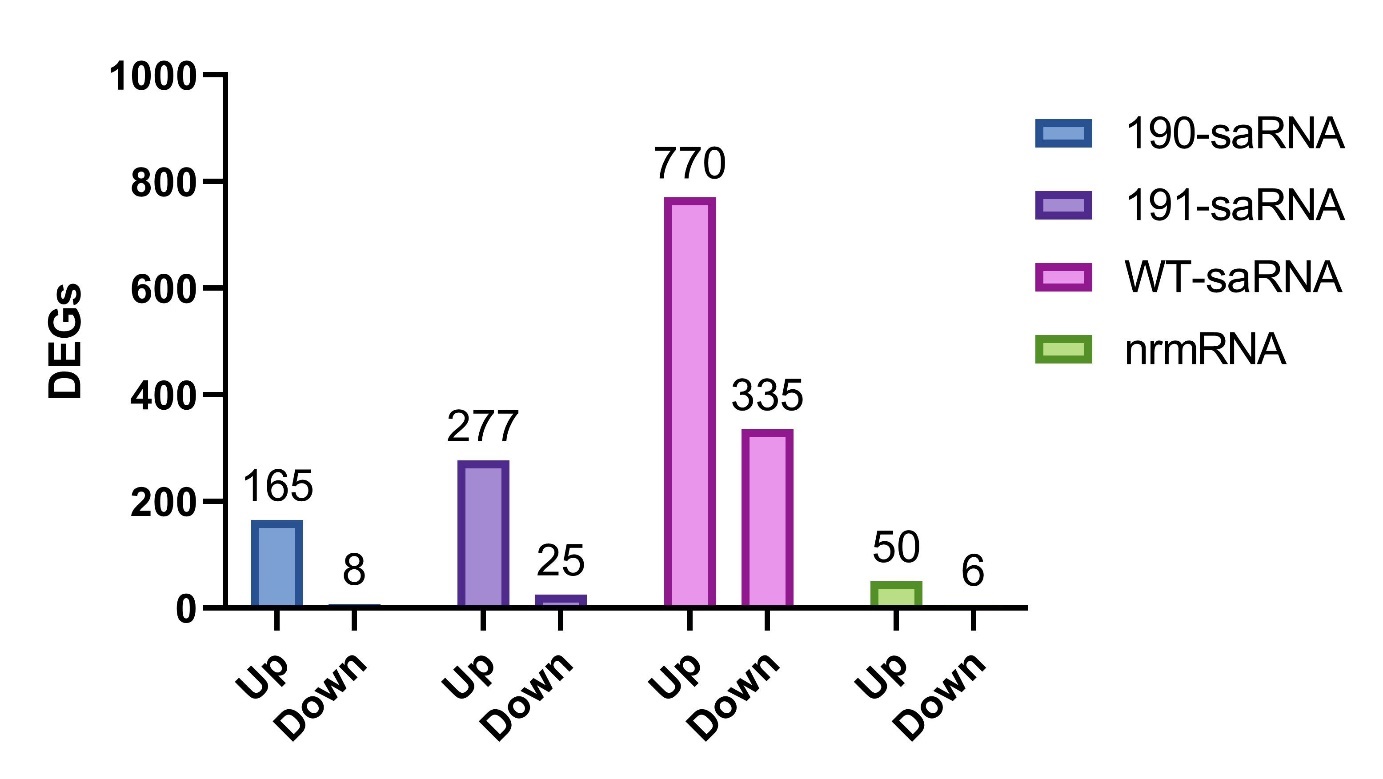


**Supplementary Figure 4.** DEGs numbers of each group. Up, upregulated; down, downregulated.

**
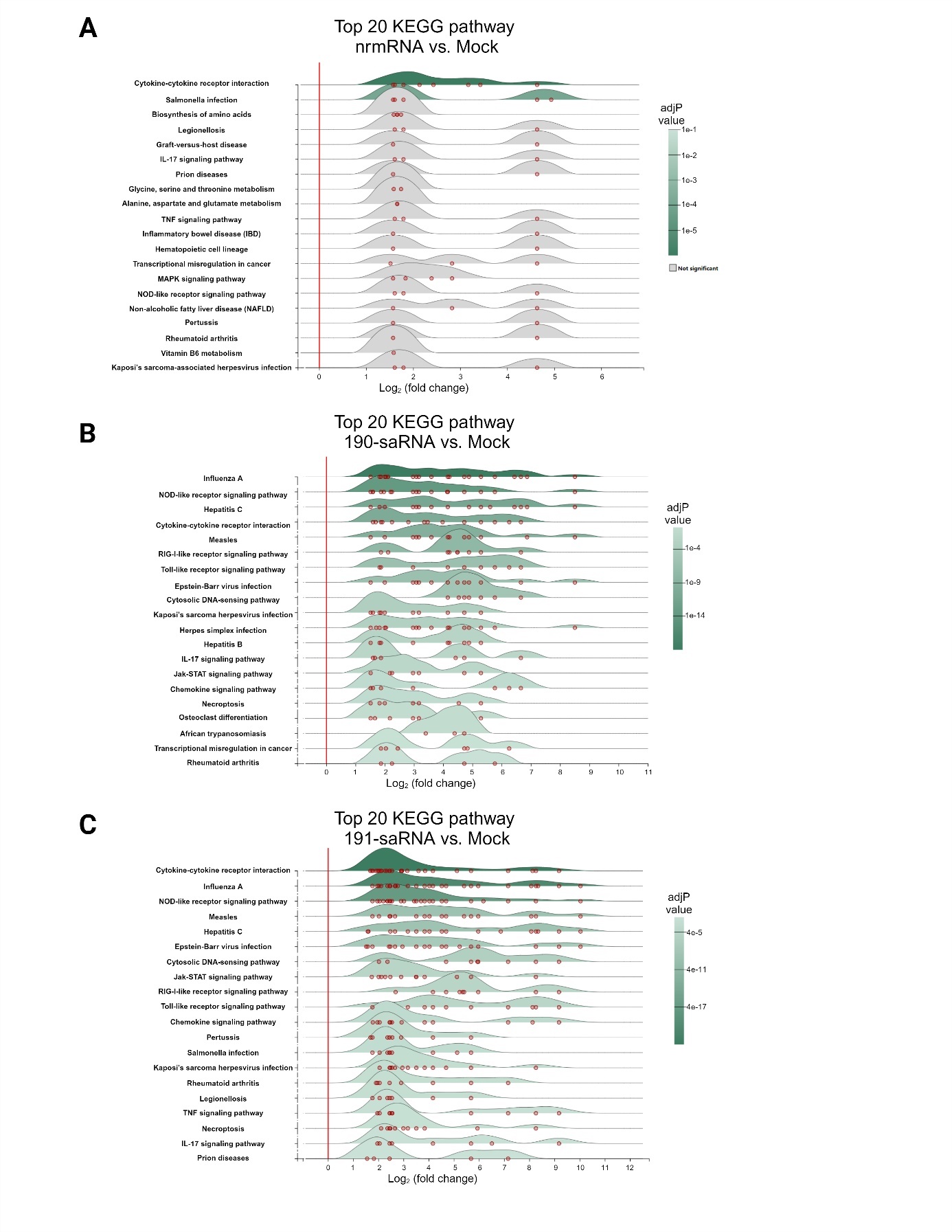
**

**Supplementary Figure 5.** KEGG enrichment analysis of different RNAs versus mutant Mock. A) Ridgeline plot of top 20 KEGG pathways enriched between differently expressed genes in non-replicating mRNA, B) 190-saRNA, or C) 191-saRNA versus Mock groups. Red dots represent each upregulated gene, and the x-axis represents the fold change of indicated genes. The darkness of green indicates the adjusted P value of each pathway or category, but the dark ridgeline indicates insignificant pathways.


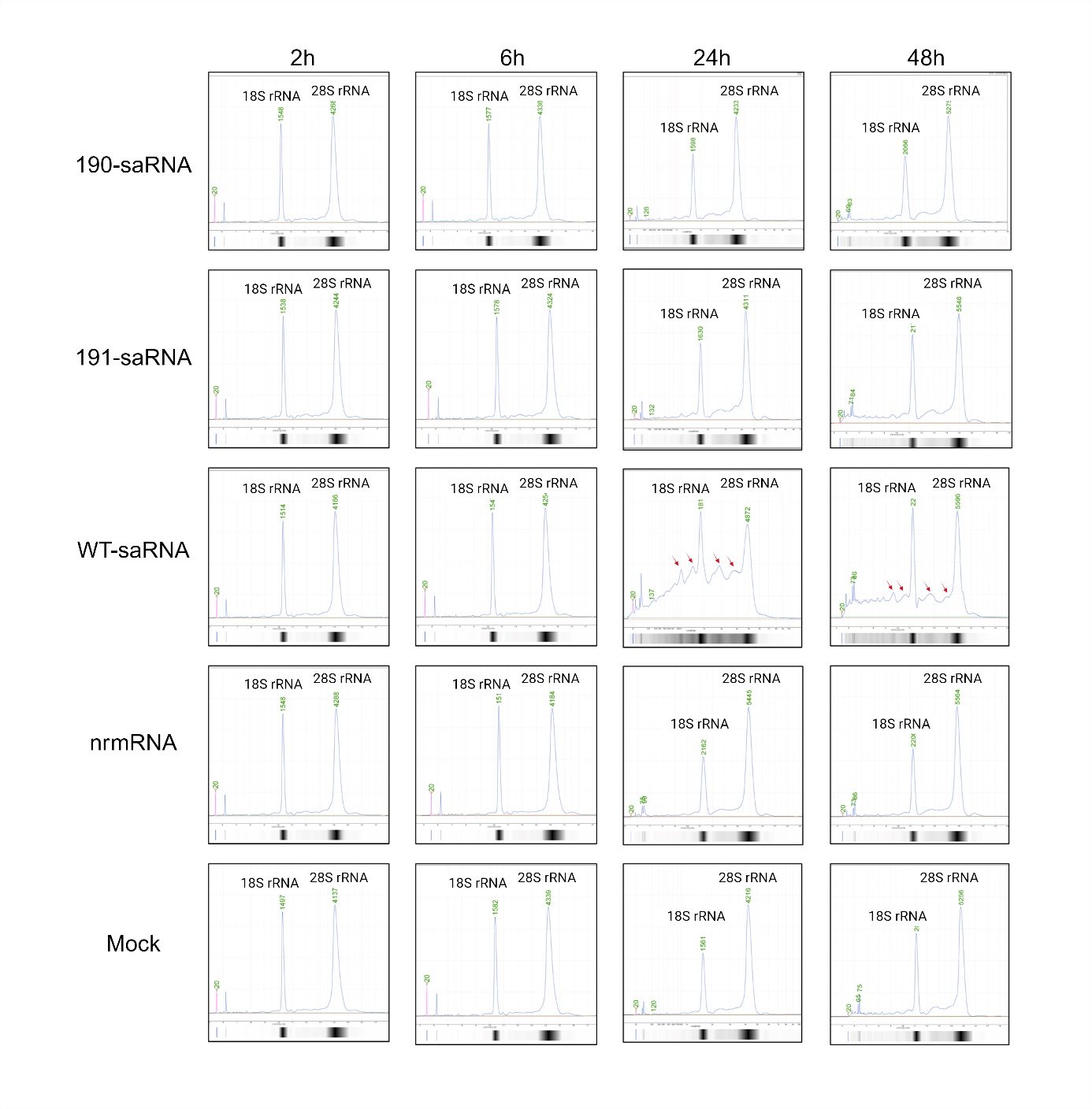


**Supplementary Figure 6.** Ribosomal RNA integrity in HeLa cells at 2, 6, 24, and 48 hours after transfection with indicated RNAs. The red arrow indicated the degraded ribosomal RNA.


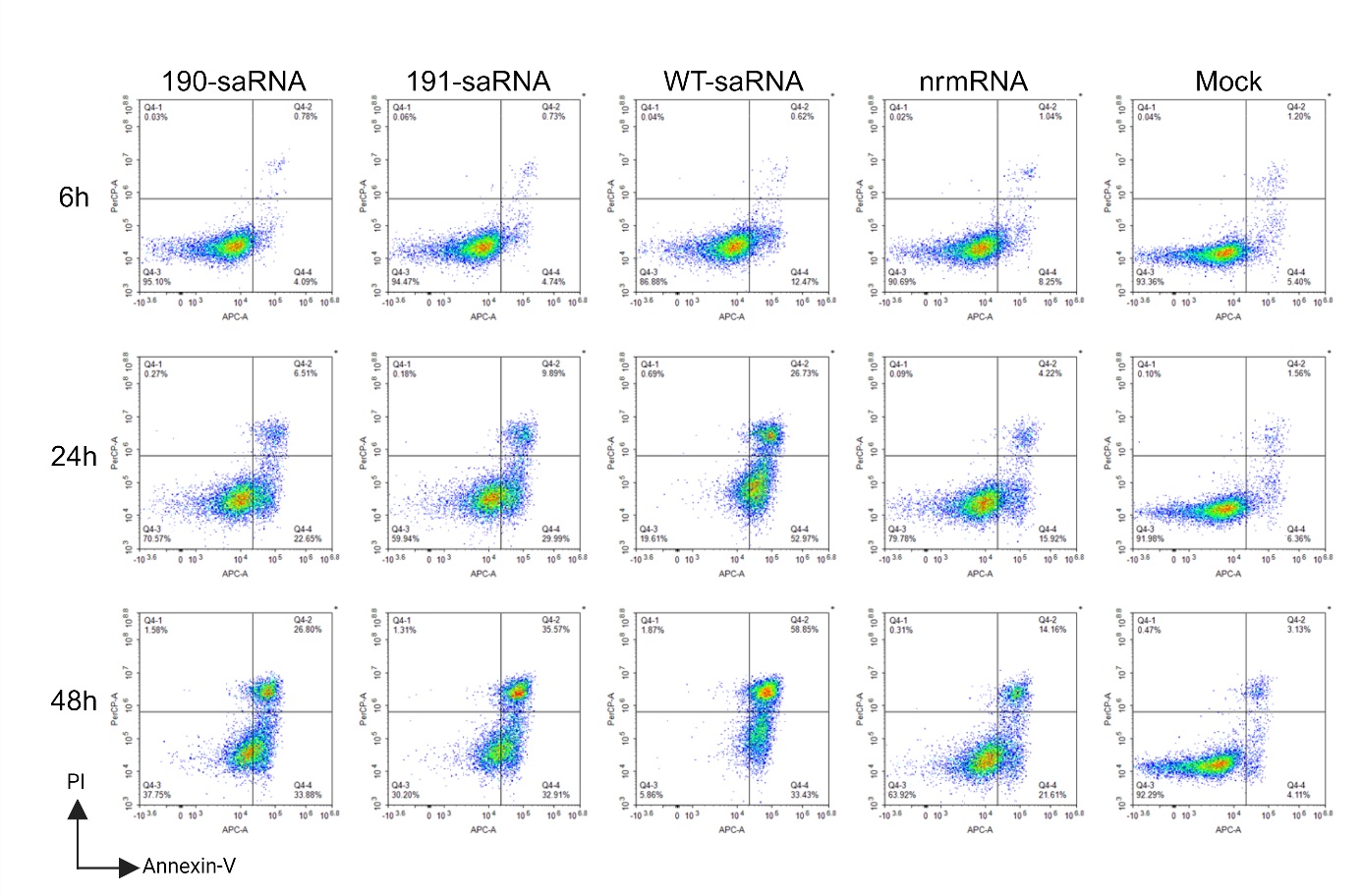


**Supplementary Figure 7.** Apoptosis analysis of HeLa cells at 6, 24, and 48 hours after transfection with indicated RNAs. The RNA-transfected cells (EGFP positive) HeLa cells were gated for apoptosis analysis, and the Mock group served as negative control.

**Supplementary Table 1.** Comparison of shared upregulated or downregulated DEGs among each group.

| Pattern | Comparison | Groups | | | |
| --- | --- | --- | --- | --- | --- |
|  |  | nrmRNA | 190-saRNA | 191-saRNA | WT-saRNA |
| Upregulated genes | Number of total upregulated genes | 50 | 167 | 277 | 770 |
|  | Number of genes common to WT- saRNA | 38 | 164 | 267 | 770 |
|  | Common gene/total gene | 38/50 | 164/167 | 267/277 | 770/770 |
|  |  | (76%) | (98.2%) | (96.39%) | (100%) |
|  | Common genes/WT-saRNA group | 38/770 | 164/770 | 267/770 | 770/770 |
|  |  | (4.94%) | (21.3%) | (34.68%) | (100%) |
|  |  |  |  |  |  |
| Downregulated genes | Number of total downregulated genes | 6 | 8 | 25 | 335 |
|  | Number of genes common to WT-saRNA | 3 | 3 | 20 | 335 |
|  | Common gene/total gene | 3/6 | 3/8 | 20/25 | 335/335 |
|  |  | (50%) | (37.5%) | (80%) | (100%) |
|  | Common genes/WT-saRNA group | 3/335 | 3/335 | 20/335 | 335/335 |
|  |  | (0.9%) | (0.9%) | (5.97%) | (100%) |

**Supplementary Table 2.** The top 20 upregulated KEGG pathway in wt saRNA versus mock group.

| Pathway | AdjP | Hits | Genes |
| --- | --- | --- | --- |
| Cytokine-cytokine receptor interaction | 3.71E-25 | 47 | IL7R, TNFSF10, CCL2, IL6, CSF1, LIF, CXCL2, IL1A, IL1RAP, FAS, IL32, IL20RB, IL15RA, CCL5, CXCL16, CXCL3, IL15, TGFB2, IL18, GDF15, CXCL8, CXCL1, IL22RA1, TNFRSF12A, INHBE, CXCL10, IFNB1, CCL8, TSLP, CXCL11, TNFSF13B, TNFRSF9, IL7, AMH, NGF, IFNL1, TNFSF13, INHBA, IL11, TNFRSF1B, BMP4, IL1B, TNFRSF8, TNFSF15, CCL7, IL23A, IL17RE |
| NOD-like receptor signaling pathway | 5.09E-20 | 44 | BIRC3, OAS1, OAS3, STAT1, GBP3, GBP1, TNFAIP3, IFI16, JUN, STAT2, RIPK2, CCL2, NAMPT, IL6, OAS2, CASP8, CXCL2, MYD88, GBP4, IRF7, NFKB1, PLCB4, CASP1, TXNIP, IRF9, NFKBIA, TRAF2, NFKBIB, PANX1, CASP4, TLR4, CCL5, GBP5, CXCL3, TICAM1, IL18, CXCL8, CXCL1, RBCK1, NEK7, IFNB1, CARD16, MEFV, IL1B |
| TNF signaling pathway | 6.22E-17 | 35 | BIRC3, TNFAIP3, IRF1, JUN, CCL2, IL6, CSF1, CASP8, MLKL, SOCS3, LIF, CXCL2, JAG1, JUNB, NFKB1, CASP7, NFKBIA, FAS, TRAF2, CCL5, ICAM1, EDN1, CXCL3, IL15, BCL3, PTGS2, TRAF1, FOS, CXCL1, CXCL10, IFNB1, CASP10, AKT3, TNFRSF1B, IL1B |
| Influenza A | 8.58E-17 | 39 | EIF2AK2, OAS1, OAS3, IFIH1, STAT1, TRIM25, PML, MX1, TLR3, STAT2, TNFSF10, CCL2, ADAR, IL6, OAS2, CASP8, SOCS3, DDX58, MYD88, IRF7, NFKB1, CASP1, IL1A, IRF9, NFKBIA, FAS, NFKBIB, JAK2, TLR4, CCL5, ICAM1, TICAM1, IL18, CXCL8, RSAD2, CXCL10, IFNB1, AKT3, IL1B |
| Kaposi's sarcoma-associated herpesvirus infection | 9.19E-12 | 36 | EIF2AK2, STAT1, TLR3, JUN, HLA-E, STAT2, IL6, CASP8, CXCL2, IRF7, NFKB1, VEGFA, IRF9, NFKBIA, ZFP36, FAS, CCND1, NFATC2, TRAF2, RCAN1, HLA-B, JAK2, FGF2, ICAM1, CXCL3, PTGS2, TICAM1, HLA-F, FOS, CXCL8, CXCL1, IFNB1, PDGFB, GNG4, AKT3, PLCG2 |
| Measles | 1.11E-10 | 29 | EIF2AK2, OAS1, OAS3, IFIH1, STAT1, TNFAIP3, MX1, JUN, STAT2, ADAR, IL6, OAS2, CASP8, DDX58, MYD88, IRF7, NFKB1, IL1A, IRF9, NFKBIA, FAS, CCND1, NFKBIB, TLR4, FOS, IFNB1, AKT3, HSPA6, IL1B |
| Epstein-Barr virus infection | 1.65E-10 | 36 | EIF2AK2, NFKB2, OAS1, OAS3, STAT1, GADD45A, TNFAIP3, TAP1, JUN, ISG15, HLA-E, STAT2, IL6, OAS2, TAP2, CASP8, DDX58, MYD88, IRF7, NFKB1, RELB, IRF9, NFKBIA, FAS, CCND1, TRAF2, HLA-B, GADD45B, NFKBIB, ICAM1, HLA-F, CXCL10, IFNB1, NFKBIE, AKT3, PLCG2 |
| Rheumatoid arthritis | 1.65E-10 | 21 | JUN, CCL2, IL6, CSF1, VEGFA, IL1A, TLR4, CCL5, ICAM1, IL15, FOS, TGFB2, IL18, CXCL8, CXCL1, TNFSF13B, TNFSF13, IL11, IL1B, ATP6V0A4, IL23A |
| MAPK signaling pathway | 2.22E-10 | 43 | NFKB2, GADD45A, DUSP1, JUN, EPHA2, DDIT3, CSF1, EREG, MYD88, DUSP5, NFKB1, EFNA1, RELB, BDNF, VEGFA, IL1A, IL1RAP, FAS, TRAF2, GADD45B, RAPGEF2, RASGRP3, FGF2, DUSP8, DUSP4, FOS, TGFB2, DUSP10, NGF, PLA2G4C, AREG, MAPK8IP2, PDGFB, AKT3, CACNA1I, CACNG8, HSPA6, IL1B, FGF21, RASGRP1, FLT3LG, DUSP6, CACNA1A |
| Osteoclast differentiation | 3.13E-10 | 26 | NFKB2, STAT1, JUN, STAT2, CSF1, SOCS3, JUNB, NFKB1, FOSL2, RELB, FHL2, IL1A, IRF9, NFKBIA, NFATC2, TRAF2, FOSL1, FOS, TGFB2, ITGB3, IFNB1, FOSB, SOCS1, AKT3, IL1B, PLCG2 |
| NF-kappa B signaling pathway | 5.31E-10 | 22 | BIRC3, NFKB2, TNFAIP3, TRIM25, CXCL2, DDX58, MYD88, NFKB1, RELB, NFKBIA, BCL10, TRAF2, GADD45B, TLR4, ICAM1, PTGS2, TICAM1, TRAF1, CXCL8, TNFSF13B, IL1B, PLCG2 |
| IL-17 signaling pathway | 1.99E-09 | 21 | TNFAIP3, JUN, CCL2, IL6, CASP8, CXCL2, NFKB1, NFKBIA, TRAF2, CXCL3, PTGS2, FOSL1, FOS, CXCL8, CXCL1, CXCL10, FOSB, MMP13, IL1B, CCL7, IL17RE |
| Legionellosis | 3.10E-09 | 17 | NFKB2, IL6, CASP8, CXCL2, MYD88, NFKB1, BCL2L13, CASP1, CASP7, NFKBIA, TLR4, CXCL3, IL18, CXCL8, CXCL1, HSPA6, IL1B |
| Pertussis | 6.41E-08 | 17 | IRF1, JUN, IL6, MYD88, NFKB1, CASP1, CASP7, IL1A, TLR4, C1R, TICAM1, FOS, CXCL8, SERPING1, C4A, IL1B, IL23A |
| Toll-like receptor signaling pathway | 1.50E-07 | 19 | STAT1, TLR3, JUN, IL6, CASP8, MYD88, IRF7, NFKB1, NFKBIA, TLR4, CCL5, TICAM1, FOS, CXCL8, CXCL10, IFNB1, CXCL11, AKT3, IL1B |
| Cytosolic DNA-sensing pathway | 1.58E-07 | 15 | ADAR, IL6, DDX58, IRF7, NFKB1, CASP1, NFKBIA, NFKBIB, CGAS, CCL5, IL18, CXCL10, IFNB1, ZBP1, IL1B |
| Jak-STAT signaling pathway | 1.58E-07 | 23 | STAT1, IL7R, STAT2, IL6, SOCS3, LIF, IRF9, CCND1, JAK2, IL20RB, IL15RA, IL15, IL22RA1, IFNB1, SOCS2, TSLP, IL7, IFNL1, SOCS1, PDGFB, IL11, AKT3, IL23A |
| Hepatitis C | 2.57E-07 | 26 | EIF2AK2, OAS1, OAS3, STAT1, MX1, CLDN1, TLR3, IFIT1, STAT2, OAS2, CASP8, SOCS3, DDX58, IRF7, NFKB1, IRF9, NFKBIA, FAS, CCND1, TRAF2, TICAM1, RSAD2, CXCL10, IFNB1, CLDN23, AKT3 |
| Pathways in cancer | 5.61E-07 | 52 | BIRC3, NFKB2, STAT1, GADD45A, PML, IL7R, JUN, STAT2, IL6, PMAIP1, CASP8, EPAS1, JAG1, NFKB1, PLCB4, DAPK3, CASP7, VEGFA, PTGER4, NFKBIA, EDNRA, FAS, CCND1, TRAF2, GADD45B, JAK2, IL15RA, RASGRP3, FGF2, EDN1, IL15, PTGS2, TRAF1, FOS, TGFB2, CXCL8, NKX3-1, IL7, PDGFB, GNG4, FZD8, AKT3, LAMA2, BMP4, BDKRB1, FGF21, RASGRP1, PLCG2, FLT3LG, WNT4, PTGER3, IL23A |
| Malaria | 9.21E-07 | 10 | THBS1, CCL2, IL6, MYD88, TLR4, ICAM1, TGFB2, IL18, CXCL8, IL1B |

**Supplementary Table 3.** The top 10 downregulated GO-biological process categories in wt saRNA versus mock group.

| Pathway | Adjust P value | Hits | Genes |
| --- | --- | --- | --- |
| Behavior | 0.332 | 20 | TMOD1, ASIC1, PDGFRB, ROBO2, KCNIP3, PRKAR2B, SCN1A, GRM1, MPP1, STRA6, PLA2G7, CHRNA3, MAPT, CCR7, OTOG, SLC1A2, NPY4R, GIP, PMCH, UCN |
| Transmission of nerve impulse | 0.623 | 23 | ASIC1, ROBO2, SCN1A, GRM1, CACNA1G, MYRF, TSNARE1, CHRNA3, PCDHB11, TRIM9, LIN7A, SYNGR1, SLC1A2, CNIH2, SYN3, YPEL1, KIF5A, GIP, PMCH, P2RX1, CHRNA9, KCNIP2, UCN |
| Synaptic transmission | 0.623 | 20 | ASIC1, ROBO2, GRM1, CACNA1G, TSNARE1, CHRNA3, PCDHB11, TRIM9, LIN7A, SYNGR1, SLC1A2, CNIH2, SYN3, KIF5A, GIP, PMCH, P2RX1, CHRNA9, KCNIP2, UCN |
| Neurological system process | 0.623 | 32 | MAP1A, ASIC1, F2R, ROBO2, KCNIP3, PRKAR2B, SCN1A, GRM1, STRA6, CACNA1G, MYRF, TSNARE1, CHRNA3, PCDHB11, ABCA4, TRIM9, PJVK, RDH5, LIN7A, OTOG, SYNGR1, SLC1A2, CNIH2, SYN3, YPEL1, KIF5A, GIP, PMCH, P2RX1, CHRNA9, KCNIP2, UCN |
| System process | 0.623 | 45 | MAP1A, TMOD1, ASIC1, MYLK3, F2R, PTGS1, ROBO2, KCNIP3, PRKAR2B, CTNNBIP1, SCN1A, GRM1, STRA6, CACNA1G, MYRF, KLHL41, TSNARE1, C1QTNF3, CALCRL, CHRNA3, PCDHB11, CFTR, ABCA4, TRIM9, PJVK, RDH5, MYOT, ELN, LIN7A, OTOG, SYNGR1, NFE2, SLC1A2, NPY4R, CNIH2, SYN3, YPEL1, KIF5A, GIP, PMCH, P2RX1, CHRNA9, KCNIP2, AQP5, UCN |
| Neurotransmitter secretion | 0.623 | 6 | ASIC1, TSNARE1, CHRNA3, TRIM9, LIN7A, SYN3 |
| Locomotory behavior | 0.623 | 7 | TMOD1, SCN1A, GRM1, CHRNA3, MAPT, OTOG, GIP |
| Feeding behavior | 0.623 | 4 | STRA6, NPY4R, PMCH, UCN |
| Secretion by cell | 0.623 | 23 | RAB26, ASIC1, F2R, PRKAR2B, LTBP2, PLCD4, GPLD1, SCAMP5, CD36, TSNARE1, C1QTNF3, CHRNA3, TRIM9, LGI3, CCR7, ITGA2B, LIN7A, NOX5, SYN3, GIP, P2RX1, KRT20, UCN |
| Homophilic cell adhesion | 0.623 | 6 | ROBO2, PCDHB11, PCDHGB7, PCDHGB1, PCDHGB4, PCDH12 |

**Supplementary Table 4.** The top 10 downregulated GO-molecular function categories in wt saRNA versus mock group.

| Pathway | Adjust P value | Hits | Genes |
| --- | --- | --- | --- |
| Calcium ion binding | 7.61E-04 | 28 | NELL2, MATN2, RHBDL3, AIF1L, KCNIP3, S100A3, LTBP2, FBLN7, ANXA9, PLCD4, CRTAC1, ADGRL4, RCN3, PROCA1, RHBDL1, PCDHB11, PLA2G3, PCDHGB7, PCDHGB1, PCDHGB4, NOX5, MAN1C1, S100A5, EFHC2, RAB11FIP4, PCDH12, KCNIP2, EFCAB5 |
| Gated channel activity | 0.287 | 11 | ASIC1, KCNIP3, SCN1A, CACNA1G, CHRNA3, KCNU1, CFTR, CLCA2, P2RX1, CHRNA9, KCNIP2 |
| G_protein coupled receptor activity | 0.287 | 12 | ADGRA3, PDGFRB, F2R, GRM1, PTH2R, ADGRL4, CALCRL, GNRHR, CCR7, SMO, NPY4R, GPR162 |
| Substrate specific channel activity | 0.287 | 13 | ASIC1, KCNIP3, SCN1A, CACNA1G, CHRNA3, KCNU1, CFTR, CLCA2, NOX5, P2RX1, CHRNA9, KCNIP2, AQP5 |
| Neurotransmitter receptor activity | 0.287 | 3 | ANXA9, CHRNA3, NPY4R |
| Glycosaminoglycan binding | 0.287 | 8 | DCN, LTBP2, FBLN7, VIT, BGN, FGFBP1, CHRD, LIPG |
| Cation channel activity | 0.335 | 10 | ASIC1, KCNIP3, SCN1A, CACNA1G, CHRNA3, KCNU1, NOX5, P2RX1, CHRNA9, KCNIP2 |
| Phospholipase activity | 0.335 | 6 | PLCD4, GPLD1, PLA2G7, PROCA1, PLA2G3, LIPG |
| Ligand gated channel activity | 0.335 | 6 | ASIC1, CHRNA3, CFTR, CLCA2, P2RX1, CHRNA9 |
| Phospholipase A2 activity | 0.335 | 3 | PLA2G7, PROCA1, PLA2G3 |

**Supplementary Table 5.** The sequences information of the primers.

| Primer | Forward or reverse | Sequence (5′ to 3′) |
| --- | --- | --- |
| GAPDH | F | GGTATCGTGGAAGGACTC |
|  | R | GTAGAGGCAGGGATGATG |
| EGFP | F | GAACCATCTTCTTCAAGGACG |
|  | R | TGGCCCAGGATGTTGC |
| TLR3 | F | GCGCTAAAAAGTGAAGAACTGGAT |
|  | R | GCTGGACATTGTTCAGAAAGAGG |
| MDA5 | F | GCTGAAGTAGGAGTCAAAGCCC |
|  | R | CCACTGTGGTAGCGATAAGCAG |
| RIG-1 | F | CACCTCAGTTGCTGATGAAGGC |
|  | R | GTCAGAAGGAAGCACTTGCTACC |
| OAS1 | F | AGGAAAGGTGCTTCCGAGGTAG |
|  | R | GGACTGAGGAAGACAACCAGGT |
| OAS3 | F | GGGCTTCTAATACCTGTGCC |
|  | R | AGGCAGTATCGATGAGGTGA |
| PKR | F | GAAGTGGACCTCTACGCTTTGG |
|  | R | TGATGCCATCCCGTAGGTCTGT |
| IFN-beta | F | GTCACTGTGCCTGGACCATAG |
|  | R | GTTTCGGAGGTAACCTGTAAGTC |
| IFIT2 | F | GGAGCAGATTCTGAGGCTTTGC |
|  | R | GGATGAGGCTTCCAGACTCCAA |
| CCL5 | F | CCTGCTGCTTTGCCTACATTGC |
|  | R | ACACACTTGGCGGTTCTTTCGG |
| CXCL10 | F | GTGGCATTCAAGGAGTACCTC |
|  | R | TGATGGCCTTCGATTCTGGATT |
| TNFα | F | CTCTTCTGCCTGCTGCACTTTG |
|  | R | ATGGGCTACAGGCTTGTCACTC |
| 198 | R | CATGGTGGCACTAGTCTTG |
| 247 | F | ATGGAGAAAGTTCACGTTGACATCGAG |
| Mouse IFNα | F | TCCATCAGCAGCTCAATGAC |
|  | R | AGGAAGAGAGGGCTCTCCAG |
